# Sequence polymorphisms in wild, weedy, and cultivated rice suggest seed-shattering locus *sh4* played a minor role in Asian rice domestication

**DOI:** 10.1002/ece3.318

**Published:** 2012-07-24

**Authors:** Yongqing Zhu, Norman C Ellstrand, Bao-Rong Lu

**Affiliations:** 1 Ministry of Education Key Laboratory for Biodiversity and Ecological Engineering, Institute of Biodiversity Science, Fudan UniversityHandan Road 220, Shanghai, 200433, China; 2 Department of Botany and Plant Sciences, Center for Conservation Biology, and Center for Invasive Species Research, University of CaliforniaRiverside, California, 92521-0124, China

**Keywords:** Crop evolution, domestication, haplotype analysis, *Oryza*, seed shattering, sequence polymorphism, *sh4*

## Abstract

The predominant view regarding Asian rice domestication is that the initial origin of nonshattering involved a single gene of large effect, specifically, the *sh4* locus via the evolutionary replacement of a dominant allele for shattering with a recessive allele for reduced shattering. Data have accumulated to challenge this hypothesis. Specifically, a few studies have reported occasional seed-shattering plants from populations of the wild progenitor of cultivated rice (*Oryza rufipogon* complex) being homozygous for the putative “nonshattering” *sh4* alleles. We tested the *sh4* hypothesis for the domestication of cultivated rice by obtaining genotypes and phenotypes for a diverse set of samples of wild, weedy, and cultivated rice accessions. The cultivars were fixed for the putative “nonshattering” allele and nonshattering phenotype, but wild rice accessions are highly polymorphic for the putative “nonshattering” allele (frequency ∼26%) with shattering phenotype. All weedy rice accessions are the “nonshattering” genotype at the *sh4* locus but with shattering phenotype. These data challenge the widely accepted hypothesis that a single nucleotide mutation (“G”/“T”) of the *sh4* locus is the major driving force for rice domestication. Instead, we hypothesize that unidentified shattering loci are responsible for the initial domestication of cultivated rice through reduced seed shattering.

## Introduction

Domestication is the anthropogenic process by which a wild species evolves into new forms that meet human needs (Doebley et al. 2006). Plant domestication has created hundreds of crops that have contributed tremendously in human civilization (Diamond 2002). A handful of agro-morphological and physiological characteristics, such as reduced seed shattering and dormancy, increased grain size, and synchronization of flowering and seed maturation, are essential for the evolution of domestication of major grain crops (Sang and Ge 2007). Consequently, this suite of characters is commonly referred to as the “domestication syndrome” (Harlan 1975). Perhaps, the most important of these characters is reduced seed shattering determined by genetic changes in the development in the abscission layers at the base of seeds or spikelets (Fuller et al. 2009). Therefore, studies elucidating the genetic basis of reduced seed shattering are critical for understanding the evolutionary process of cereal crop domestication.

**Figure 1:**
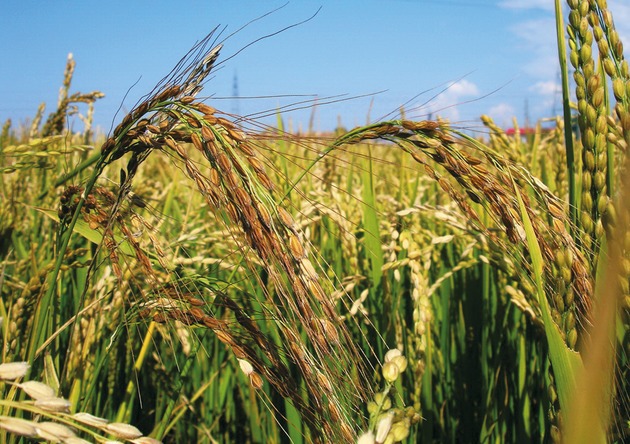
Panicles of cultivated rice (*Oryza sativa*, back) and weedy rice (*O. sativa* f. *spontanea*, front) that co-occurs with in agricultural ecosystems. Owning to its strong seed shattering at maturity, weedy rice causes a considerable yield loss of the crop rice.

**Figure 2:**
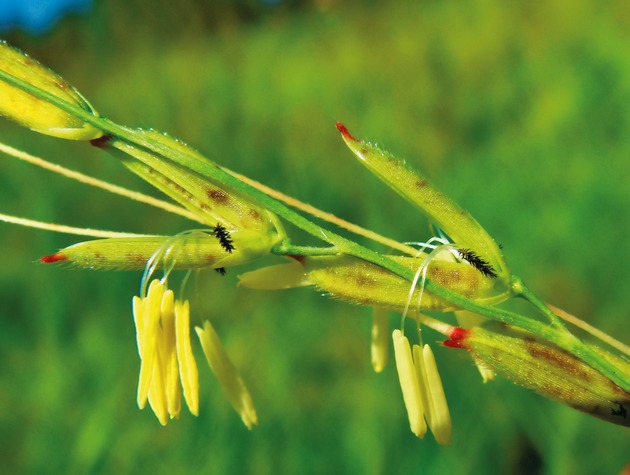
Spikelets of perennial common wild rice (*Oryza rufipogon*). This grass species with strong seed shattering is considered the direct ancestor of Asian cultivated rice domesticated ∼8000 years ago (Normile 1997; Zong et al. 2007). The change from seed shattering of a wild species to seed persistence is the key process in crop domestication.

Asian cultivated rice (*Oryza sativa* L., Fig. 1) is the world's most important cereal crop, providing the staple food for more than one half of the global population (Khush 2005). It is also one of the earliest domesticated crops. Archeological evidence suggests that domestication of *O. sativa* from wild *O. rufipogon* Griff. (Fig. 2) occurred in the middle and lower Yangzi River regions of China about 8000 years ago (Normile 1997; Zong et al. 2007).

As for other grains, a key evolutionary change during rice domestication involves the reduction in seed shattering. The infructescences of both Asian rice's wild ancestor (*O. rufipogon* complex, including the annual *O. nivara*) (Vaughan et al. 2008) and Asian rice's weedy descendant (“weedy rice” also referred to as “red rice”, *Oryza sativa* f. *spontanea*, Fig. 1) shatter easily to disperse their seeds. Human selection for seeds that not dispersed, but retained on the plant, was essential for increasing grain yields of hand-harvested rice.

The genetic basis of seed shattering in rice has been examined in studies that identified the co-segregation of the trait and molecular genetic markers in progenies descended from crosses between cultivated rice and its wild relatives. Such studies have identified several quantitative trait loci (QTL) that play a role in whether rice seeds shatter from or persist on infructescences, such as *sh3*, *sh4*, *sh6*, *sh8*, *qsh1*, *qsh2*, *qsh5*, *qsh11*, *qsh12*, and *sh-h* (Konishi et al. 2006; Li et al. 2006; Ji et al. 2010).

Two shattering QTL, *sh4* located on chromosome 4 and *qsh1* located on chromosome 1, often considered to be critical for rice domestication and evolution (Doebley et al. 2006), have been cloned (Konishi et al. 2006; Li et al. 2006). The *sh4* locus that explains as much as 69% of phenotypic variance in shattering (Li et al. 2006) was identified based on crosses between cultivated rice and wild relatives, including its ancestors in the *O. rufipogon* complex. Sequence data showed a single nonsynonymous substitution [a nucleotide substitution of “G” to “T” or an amino acid substitution of asparagine for lysine (Li et al. 2006)] in the first exon of *sh4* that co-varied with the nonshattering phenotype. Transformation experiments by Li et al. (2006) demonstrated that this substitution “undermined the gene function necessary for the normal of the abscission layer that controls the separation of a grain from the pedicel”, converting rice plants from shattering to a considerable degree of seed persistence (Li et al. 2006). This “G” to “T” substitution has been found to be fixed in all rice cultivars examined to date (Li et al. 2006; Lin et al. 2007; Zhang et al. 2009; Thurber et al. 2010). Therefore, *sh4* is widely considered to be the most significant shattering gene that is responsible for the domestication of rice from its wild ancestor (Li et al. 2006; Purugganan and Fuller 2009).

The other locus, *qsh1*, was identified in an F_2_ population derived from a cross between an *indica* rice cultivar and a *japonica* rice cultivar and explained almost 69% of phenotypic variance in shattering in that population (Konishi et al. 2006). Again, sequencing data also showed a single nonsynonymous substitution (“G” to “T”), in this case, in the 5′ upstream regulatory region of the locus that further reduced shattering ability in a subset of *japonica* rice cultivars (Konishi et al. 2006). However, the evolutionary interpretation of QTL depends on the relationship of the parents in the cross that generates the segregating population (Miles and Wayne 2008). In the case of *qsh1*, the polymorphism only involves seed-shattering variation within cultivated rice. As acknowledged by those who cloned it (Konishi et al. 2006), this allele could not play a role in rice evolution until after the early stages of domestication (Konishi et al. 2006). Indeed, the role of *qsh1* in rice evolution is now considered to be important during the improvement of *japonica* varieties, rather than evolution during the first steps of the evolution of domestication rice (Konishi et al. 2006; Thurber et al. 2010).

The current view then is that the initial origin of nonshattering during the early rice domestication involved a single gene of large effect, specifically, the *sh4* locus, with the evolutionary replacement of a dominant allele for shattering with a recessive allele for nonshattering. To date, most published sequence data of the *sh4* locus are consistent with that hypothesis involving the replacement of the wild type, with the seed-shattering “G” nucleotide, by the mutational type, with nonshattering “T” nucleotide, at *sh4*'s functional nucleotide polymorphism (FNP) site as the key event in the domestication process of Asian cultivated rice (Li et al. 2006; Lin et al. 2007; Zhang et al. 2009; Thurber et al. 2010).

However, some data challenge that hypothesis. A few studies have reported that some accessions of Asian rice's wild ancestor (*O. rufipogon*) contain the *sh4* “T” substitution despite their seed-shattering phenotype (Izawa 2008; Thurber et al. 2010). Furthermore, highly shattering weedy rice in the United States, a descendant of cultivated rice, appears to be fixed for the cultivars' “T” type (Thurber et al. 2010). Finally, the phenotypic strength of cultivated rice's *sh4*'s genotype varies almost fivefold (from as little as 15% to as much as 69% of phenotypic variation) depending on the parents used for QTL analysis (Cai and Morishima 2000; Li et al. 2006).

Collectively, these data suggest that this mutant allele at the *sh4* locus did not necessarily play a significant role in the evolution of reduced shattering in the early domestication of cultivated rice. Clearly, the *sh4* polymorphism plays some role in shattering, but it is not at all clear that it played the major role for the domestication of Asian cultivated rice. The *sh4* locus hypothesis depends on the rare mutant “T” allele replacing wild “G” allele as the earliest step in the domestication of rice. If the *sh4* locus hypothesis is correct, then given the extreme rarity of nonshattering phenotype in wild populations (Thurber et al. 2010), the recessive allele should be ≪1% in frequency in wild populations and, when homozygous, should express a nonshattering phenotype.

But we posit instead that one or more other loci contributed significantly to preventing shattering in wild rice in its early transition to domesticated rice. If our hypothesis is correct, then we would expect that populations of rice's wild ancestor should be polymorphic for *sh4*'s genotypes, that the “T” allele should not be extremely rare, because other loci also play significant roles in the expression of shattering and nonshattering. However, prior studies have not surveyed the *O. rufipogon* complex sufficiently to characterize the *sh4* polymorphism. Thus, further investigation is necessary involving *sh4* screening of a sufficient number of wild rice samples of diverse geographic origins.

We tested the hypothesis of the *sh4* locus as key to the domestication of cultivated rice by analyzing the *sh4* sequences of a large number of diverse wild, weedy, and cultivated rice samples. We identified those wild and weedy rice samples with strong seed shattering that had the putative “nonshattering” *sh4* genotype (“T” nucleotide at the FNP site). Our primary objectives were to answer the following questions: (1) Is the *sh4* “wild-type” allele (i.e., with the “G” nucleotide at the FNP site) fixed or nearly fixed in wild and weedy rice accessions? (2) If *sh4* is polymorphic in wild and weedy rice accessions, how do the “G” genotype and “T” genotype covary with the seed-shattering phenotypes of wild, weedy, and cultivated rice? Answering these two questions will allow us to address the larger question of whether the *sh4* shattering locus has played a significant role in the domestication of Asian cultivated rice.

## Materials and Methods

### Sample collection and phenotypic characterization

Our germplasm panel consisted of a total of 76 wild rice accessions, 165 weedy rice accessions, and 125 cultivated rice accessions widely collected from Asia, southern Europe, and North America (Table 1). We randomly chose one individual to represent each accession. In addition, we supplemented our data with the published DNA sequences of *sh4* from 90 wild rice accessions, 57 weedy rice accessions, and 67 cultivated rice accessions (Li et al. 2006; Zhang et al. 2009; Thurber et al. 2010). The complete list of all the accessions including our own collection and published data used is presented in Appendix S1.

**Table 1:** Genotypic variation at the functional nucleotide site of the *sh4* locus in wild, weedy, and cultivated rice samples and associated seed-shattering/persistence phenotype

Taxon	No. of sequences examined (%)^2^	Phenotype of seed shattering/persistence^3^	Genotype at the functional nucleotide site 4
*Oryza rufipogon*	122 (73.5)	Seed shattering	G
Complex^1^	44 (26.5)	Seed shattering	T
Weedy rice (*O. sativa* f. *spontanea*)	222 (100)	Seed shattering	T
Cultivated rice (*O. sativa*)			
*indica*	53 (100)	Seed persistence	T
*japonica*	139 (100)	Seed persistence	T

1 Including the perennial *Oryza rufipogon* and the annual *O. nivara* following the classification of Oka (1988).

2 Including published sequences, for detailed information see Appendix S1.

3 Detailed information on phenotypes of individual samples is presented in Appendix S1.

4 Detailed information on “G” versus “T” genotype is presented in Appendix S1.

The phenotypes of seed shattering versus persistence were determined by gently gripping each of three naturally mature panicles per plant by hand. If more than 60% seeds were released from each panicle by this treatment, a plant was determined to have a “seed-shattering” phenotype; if less, the phenotype was characterized as “seed persistence”.

### DNA extraction, polymerase chain reaction, and sequencing

DNA was extracted from leaf tissues of seedlings germinated from seeds grown in a greenhouse. The amplified region of *sh4* included the entire first exon (847 bp) and a partial intron (153 bp) of *sh4* gene and a 5′ upstream flanking region (1509 bp) (Zhang et al. 2009). The DNA amplifying system followed that of Zhang et al. (2009). The internal sequencing primers were designed using the Primer3Plus (Rozen and Skaletsky 2000), based on rice genome sequences (Feng et al. 2002; Matsumoto et al. 2005). For the obligately outcrossing wild rice, the purified polymerase chain reaction (PCR) products were cloned into pMD18-T vectors (Takara, Dalian, China); eight randomly selected clones per seedling were sequenced. For highly selfing weedy and cultivated rice, the purified PCR products were directly sequenced in both directions. Singletons and ambiguous sites were re-sequenced to assure the quality of sequences used for analyses. All DNA sequences obtained for this study were deposited in GenBank (accession numbers: JN679233–JN679598).

### Sequence analyses

Sequences of each rice accession were assembled by Seqman II (DNASTAR, Madison, Wisconsin), and the ends of amplicons were trimmed to remove low-quality sequences. Sequence alignments were performed using ClustalX (Thompson et al. 1997) and were refined manually. All aligned sequences were imported into the DnaSP package (Librado and Rozas 2009) to extract all the nucleotide polymorphic sites on which our various haplotypes were identified. The FNP sites (“G” vs. “T”), generally considered to be responsible for rice's seed shattering/persistence, were identified for all sequences and compared among wild, weedy, and cultivated rice accessions.

## Results

### Wild, weedy, and cultivated rice seed-shattering/persistence phenotypes and *sh4* FNP

**Figure 3:**
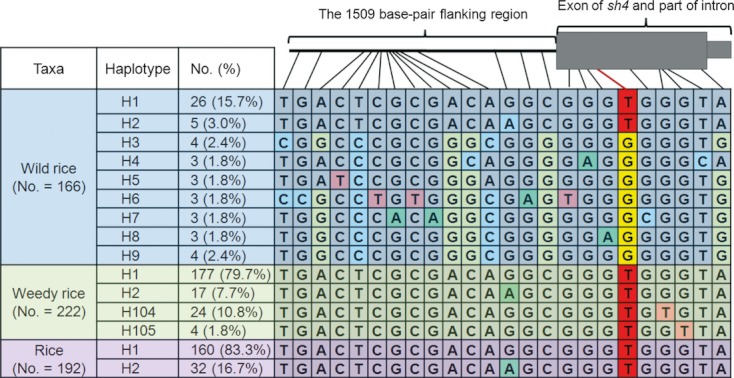
Nucleotide variation (color coded) of selected (frequency >1.5%) haplotypes in the sequenced region**.** All cultivated rice, weedy rice, and some wild rice accessions have the “T” nucleotide at the functional nucleotide polymorphism (FNP) site (highlighted with red). Haplotypes H1 and H2 are shared by wild, weedy, and cultivated rice samples. H3 to H9 were only detected in wild samples; H104 to H105 were only found in weedy samples. *N*, number of samples, haplotype frequencies in parentheses.

All 166 wild rice accessions examined from more than a dozen Asian countries had a seed-shattering phenotype; likewise all 222 geographically diverse weedy rice accessions from many countries of three continents had the same seed-shattering phenotype (Table 1, Appendix S1). We examined the DNA sequences of all the 166 accessions of wild rice and 222 accessions of weedy rice from a wide geographic range to determine whether the FNP site of *sh4* locus was the wild-type (putatively, seed shattering) “G” nucleotide or the mutational type (putatively, nonshattering) “T” nucleotide (Appendix S1). We found considerable polymorphism in the wild rice samples. Almost three-quarters (73.5%) of the wild rice accessions contained the wild-type “G” nucleotide at the FNP site, whereas a sizeable minority (26.5%) of the wild accessions had the nonshattering “T” nucleotide (Fig. 3, Table 1, Appendix S2). Interestingly, all (100%) weedy rice accessions examined had the inappropriate “T” nucleotide (Fig. 3, Table 1, Appendices S1 and S2), although every weedy rice accession showed the seed-shattering phenotype (Table 1, Appendix S1). Thus, we found no relationship between presence of FNP “G” nucleotide or the “T” nucleotide and the seed-shattering/persistent phenotypes of wild and weedy rice. All 192 cultivated rice accessions, including both *indica* and *japonica*, examined were found to have the seed persistence phenotype (Table 1, Appendix S1).The sequencing data showed that all cultivated rice accessions examined had the “T” nucleotide at *sh4*'s FNP (Fig. 3, Table 1, Appendices S1 and S2).

### *Sh4* locus and flanking region sequence polymorphisms in wild, weedy, and cultivated rice

We compared the sequences of the entire first exon of *sh4* and about 1500 bp of its upstream flanking region in our wild, weedy, and cultivated rice accessions. We found significant sequence polymorphism in wild rice accessions (Appendix S2), where a total of 101 variable sites were identified. Based on these variable sites, we determined a total of 103 haplotypes for 166 wild rice accessions (Table 2, Appendix S3). In contrast, much less polymorphism was detected in the target sequences of weedy and cultivated rice accessions, with only one to three variable sites identified. We determined four haplotypes for weedy rice accessions and only two haplotypes for cultivated rice accessions (including *indica* and *japonica* rice). The haplotype frequencies in wild, weedy, and cultivated rice, as well as haplotype origins and associated shattering phenotypes, are provided in Appendix S3.

**Table 2:** Nucleotide diversity and number of haplotypes of the examined DNA sequences of the *sh4* locus in wild, weedy, and cultivated rice accessions

Taxon	No. of sequences examined	No. of variable sites identified	No. of haplotypes identified
*Oryza rufipogon* complex^1^	166	101	103
Weedy rice (*O. sativa* f. *spontanea*)	222	3	4
Cultivated rice (*O. sativa*)	192	1	2

1 Including the perennial *Oryza rufipogon* and the annual *O. nivara* following the classification of Oka (1988).

For wild rice, nine “common” haplotypes, each represented by more than three accessions, accounted for 32.5% of all wild rice accessions (Appendix S3). The 94 “rare” haplotypes, those represented by only 1–2 accessions, accounted for the rest, 67.5% of wild rice accessions (Table 2, Appendix S3). Of the nine “common” haplotypes, about 19% of the total wild rice accessions were identified to as haplotype-1 (H1) or haplotype-2 (H2) (Fig. 3) differing from one another by a single nucleotide substitution (“G” to “A”) at 1158 bp site of the flanking region (Appendix S2). About 14% wild rice accessions were the haplotype-3 to -9 (H3–H9) (Appendix S3).

In the 222 weedy rice accessions, only four haplotypes (H1, H2, H104, and H105) were identified. About 87% of the total weedy rice accessions were identified to be H1 or H2. Two weedy haplotypes were unique to specific regions; 24 weedy rice accessions from Italy and Spain were identified as H104, and four weedy accessions from the USA had H105 (Table 2, Appendix S3).

In the 192 cultivated rice accessions, only two haplotypes (H1 and H2) were identified. H1 was represented by the majority (83.3%) of cultivated accessions. H2 was present in the minority (16.7%) (Table 2, Appendix S3).

Thus, H1 and H2 are not only shared by wild rice, weedy rice, and cultivated rice; they are also the most prominent haplotypes for each. Interestingly, although all of the wild and weedy rice accessions were seed shattering, and all cultivated rice accessions were seed persistent, all wild, weedy, and cultivated accessions of H1 and H2 carried the mutational type of the “T” nucleotide at the FNP site of *sh4*, regardless of their seed dispersal phenotype. All other wild rice accessions (i.e., with haplotypes other than H1 or H2) carried the wild-type “G” nucleotide at the FNP site of *sh4* (Fig. 3). The weedy rice accessions of H104 and H105 carried the mutational type “T” nucleotide at the FNP site (Fig. 3), even though these accessions were seed shattering.

## Discussion

Our data demonstrate that some seed-shattering *O. rufipogon* and all seed-shattering weedy rice accessions have the “nonshattering” genotype with the “T” nucleotide at the FNP site of *sh4*, which challenges the widely accepted hypothesis that human selection for the “T” nucleotide (and selection against the “G” nucleotide) at the *sh4* locus was a significant early step in the process of the domestication of Asian rice (Li et al. 2006; Zhang et al. 2009). In our large and diverse samples including those from published data (Li et al. 2006; Zhang et al. 2009; Thurber et al. 2010), we found considerable discordance between *Oryza* seed-shattering/persistence phenotypes and putative “nonshattering” “T” and “shattering” “G” genotypes. Specifically, we found the “T” genotype at a remarkably high frequency (nearly 27%) in shattering wild rice (*O. rufipogon* complex) accessions collected over a wide geographic area. While our screening is the numerically largest and geographically broadest to date, it supplements previous reports in which some wild rice (*O. rufipogon*) samples were also found to contain “T” nucleotide at the of *sh4* locus (Izawa 2008; Thurber et al. 2010). In fact, Li et al. (2006) who first reported the data suggesting a key role for *sh4* in rice domestication also noted three accessions of nonshattering wild rice that contained the “T” nucleotide at the FNP site, the same as cultivated rice. The authors' explanation was that these wild rice samples might be misidentified nonshattering weedy (not wild) rice that acquired the *sh4* allele from its cultivated rice ancestor. Subsequently, Zhang et al. (2009) found two wild rice samples heterozygous for the “T” and “G” nucleotides at the FNP site of *sh4*, which they explained as “most likely the result of introgression of the nonshattering allele from cultivars into the wild populations.” Our results from a large and geographically diverse set of samples are in accord with these previous reports of *sh4* locus “G”/”T” polymorphism in wild rice populations. The so-called wild-type allele is far from fixation; the “T” allele is relatively common in wild rice populations. Thus, our results do not support the *sh4* hypothesis because our large and geographically diverse set of wild rice samples we screened had a seed-shattering phenotype, independent of whether they were homozygous for either type of *sh4* allele (Appendix S1).

Interestingly, the sequence data for an even larger number and geographically more diverse of weedy rice accessions revealed that they were fixed for one allele, the “nonshattering” “T”-type of *sh4* allele, despite the fact that all of those weedy rice plants had strongly seed-shattering panicles! Thurber et al. (2010) reported the same conclusion for a few dozen weedy rice accessions in the United States. All had seed-shattering panicles, but all had the “inappropriate” allele. Furthermore, Thurber et al. (2010) also found eight seed-shattering individuals of the *O. rufipogon* complex with the nonshattering “T” allele. They made a conclusion, “the presence of this mutation alone is not sufficient to confer a reduction in shattering”. We agree. Collectively, these results strongly indicate that the presence or absence of wild “G”-type homozygosity of the *sh4* allele does not affect the seed-shattering phenotype of wild and weedy rice. In other words, the data do not support an important role for the *sh4* locus in the evolutionary shift from seed shattering to seed persistence during the domestication of cultivated rice from its wild ancestor.

Interestingly, about 19% of the phenotypically shattering wild rice accessions have the same H1 and H2 haplotypes (both having the “T”-type *sh4* variant) as all cultivated rice accessions included in our study. These results suggest that cultivated rice was most likely domesticated from wild rice sharing the H1 and H2 haplotypes. Thus, one or more loci other than *sh4* must have first conferred the shattering/persistent trait during the domestication of cultivated rice. Alternatively, introgression may also produce the same H1 and H2 haplotypes of wild and cultivated rice. However, the frequency of wild-crop introgression or recombination within such a small piece of DNA fragment (<1000 bp) within the *sh4* locus should be extremely low.

In conclusion, our data and those from prior studies (Li et al. 2006; Thurber et al. 2010) challenge the widely accepted hypothesis that the evolutionary replacement of the wild type of “G” allele by the “T” allele at FNP site of *sh4* in wild rice populations was a substrate for human-favored selection for seed persistence as the major driving force for rice domestication. The considerable polymorphism we observed for the “G” allele and “T” allele in the seed-shattering wild rice plants from different populations does not support that hypothesis. In addition, the detection of the “T” allele in a large number of diverse accessions of seed-shattering weedy rice plants also rejects that conclusion.

We hypothesize instead that the *sh4* “T” allele may have evolved to fixation in cultivated rice after the first steps of rice domestication in a role as modifier allele to an as-yet unidentified anti-shattering allele or alleles fixed at one or more other loci. Our hypothesis is consistent with the fact that the *sh4* “T” allele co-segregates with nonshattering to a variable extent in certain QTL progenies, that the locus is expressed in the appropriate abscission tissues, and that cultivated rice transformed with the “G” allele has some increase in shattering (Li et al. 2006). Recently, Zhou et al. (2012) identified a seed *shattering abortion1* (*shat1*) mutant in a wild rice introgression line (SL4). This gene is proven to encode a transcription factor that affects seed abscission zone development, and therefore, also determines rice seed shattering. After comparing the DNA sequences of wild and cultivated rice at this locus (sequences data from GenBank), we found no sequence polymorphism in the examined sequences as described by Zhou et al. (2012). We think that *shat1* should not be the unidentified locus responsible for the domestication of cultivated rice either. Further research is necessary to identify the original loci and alleles responsible for the initial reduction in shattering during the first steps of rice domestication.

Thus, we hypothesize that an unidentified locus or loci are responsible for the domestication of cultivated rice through reduced seed shattering. Once the alleles were fixed, modifier alleles at other loci were selected to further decrease seed shattering. The “T” allele at the FNP site of *sh4* is likely to be one of these that were incorporated late in the domestication process. We hypothesize that decreased shattering expression due to the “T” allele can only occur in the presence of an appropriate genotype or genotypes at other loci that were previously selected during domestication. The allele at *qsh1*, mentioned in the Introduction, is another example of an allele that further reduces seed shattering and appears to have been selected by mechanical threshing long after domestication during the crop improvement phase of temperate *japonica*.

The evolutionary process of rice domestication is filled with mysteries yet to be examined. Identifying the unknown alleles associated the origin and genetic mechanisms of domestication is important not only for cultivated rice but also for understanding those processes in other crops. Likewise, that information has practical applications, including the proper management of weedy rice whose persistence in rice fields through seed shattering causes significant rice yield losses (Delouche et al. 2007). For example, silencing the allele*-*based seed-shattering expression using RNAi biotechnology as suggested by Gressel and Valverde (2009) may mitigate the agronomic or ecological impacts caused by transgene flow to wild and weedy rice populations. Silencing the expression of the true shattering allele(s) should significantly reduce seed shattering in weedy plants that have acquired transgenes by gene flow, and consequently reduce their seed dispersal and fitness (Gressel and Valverde 2009).

## Supporting Information

**Appendix S1.** Plant materials examined in this study, including their country of origin, seed shattering phenotype, and nucleotide at the functional nucleotide polymorphism (FNP) site of the sh4 locus.

**Appendix S2.** Aligned DNA sequences of all the 105 haplotypes of the sh4 locus detected in wild, weedy and cultivated rice.

**Appendix S3.** Haplotypes detected in wild, weedy and cultivated rice with their geographic origin and seed shattering phenotype.
