# Supplementary material for: Sequence polymorphisms in wild, weedy, and cultivated rice suggest seed-shattering locus sh4 played a minor role in Asian rice domestication
Source: Ecol Evol. 2012 Jul 24;2(9):2106–13. doi: 10.1002/ece3.318 (PMC3488663; doi:10.1002/ece3.318)
Supplement: Supplementary file 1 [file ece30002-2106-SD1.doc]

| **Appendix 1. Plant materials examined in this study, including their country of origin, seed shattering phenotype, and nucleotide at the functional nucleotide polymorphism (FNP) site of the sh4 locus** | | | | | |
| --- | --- | --- | --- | --- | --- |
| **Group** | **Accession ①** | **Country or origin** | **No. accession ②** | **Seed shattering/persistance trait** | ***sh4* ③** |
| **Wild rice** | **93287** | **Nepal** | **JN679233** | **Seed shattering** | **T** |
| **(Oryza rufipogon** | **100588** | **Taiwan, China** | **JN679234** | **Seed shattering** | **T** |
| **complex)** | **100597** | **Taiwan, China** | **JN679235** | **Seed shattering** | **T** |
|  | **100599** | **Taiwan, China** | **JN679236** | **Seed shattering** | **T** |
|  | **105738** | **Cambodia** | **JN679237** | **Seed shattering** | **T** |
|  | **105763** | **Thailand** | **JN679238** | **Seed shattering** | **T** |
|  | **105767** | **Thailand** | **JN679239** | **Seed shattering** | **T** |
|  | **106075** | **India** | **JN679240** | **Seed shattering** | **T** |
|  | **106086** | **India** | **JN679241** | **Seed shattering** | **T** |
|  | **106087** | **India** | **JN679242** | **Seed shattering** | **T** |
|  | **106153** | **Laos** | **JN679243** | **Seed shattering** | **T** |
|  | **106156** | **Laos** | **JN679244** | **Seed shattering** | **T** |
|  | **106343** | **Myammar** | **JN679245** | **Seed shattering** | **T** |
|  | **Lao5-3** | **Laos** | **JN679246** | **Seed shattering** | **T** |
|  | **100588** | **Taiwan** | **JN679247** | **Seed shattering** | **T** |
|  | **104479** | **Thailand** | **JN679248** | **Seed shattering** | **T** |
|  | **104650** | **Thailand** | **JN679249** | **Seed shattering** | **T** |
|  | **100588** | **Taiwan** | **GU220980*** | **Seed shattering** | **T** |
|  | **100904** | **Thailand** | **GU220981*** | **Seed shattering** | **T** |
|  | **104833** | **Thailand** | **GU220987*** | **Seed shattering** | **T** |
|  | **106086** | **India** | **GU220996*** | **Seed shattering** | **T** |
|  | **106169** | **Vietnam** | **GU221002*** | **Seed shattering** | **T** |
|  | **106163** | **Laos** | **GU220049*** | **Seed shattering** | **T** |
|  | **86662** | **Thailand** | **GU221008*** | **Seed shattering** | **T** |
|  | **103821** | **China** | **GU221009*** | **Seed shattering** | **T** |
|  | **105742** | **Cambodia** | **EU999943*** | **Seed shattering** | **T** |
|  | **93285** | **Nepal** | **JN679250** | **Seed shattering** | **T** |
|  | **103836** | **Bangladesh** | **JN679251** | **Seed shattering** | **T** |
|  | **103838** | **Bangladesh** | **DQ383377*** | **Seed shattering** | **T** |
|  | **105894** | **Bangladesh** | **DQ383392*** | **Seed shattering** | **T** |
|  | **106185** | **India** | **DQ383393*** | **Seed shattering** | **T** |
|  | **100597** | **Taiwan, China** | **JN679252** | **Seed shattering** | **G** |
|  | **106357** | **Myammar** | **JN679253** | **Seed shattering** | **G** |
|  | **106359** | **Myanmar** | **JN679254** | **Seed shattering** | **G** |
|  | **106386** | **Myanmar** | **JN679255** | **Seed shattering** | **G** |
|  | **106081** | **India** | **JN679256** | **Seed shattering** | **G** |
|  | **106087** | **India** | **JN679257** | **Seed shattering** | **G** |
|  | **104501** | **India** | **GU220983*** | **Seed shattering** | **G** |
|  | **105804** | **Thailand** | **JN679258** | **Seed shattering** | **G** |
|  | **105805** | **Thailand** | **JN679259** | **Seed shattering** | **G** |
|  | **BL190** | **Guangdong, China** | **JN679260** | **Seed shattering** | **G** |
|  | **106266** | **Papua New Guinea** | **JN679261** | **Seed shattering** | **G** |
|  | **106267** | **Papua New Guinea** | **JN679262** | **Seed shattering** | **G** |
|  | **106253** | **Indonesia** | **JN679263** | **Seed shattering** | **G** |
|  | **104714** | **Thailand** | **GU220986*** | **Seed shattering** | **G** |
|  | **106453** | **Indonesia** | **GU221005*** | **Seed shattering** | **G** |
|  | **105888** | **Bangladesh** | **GU220995*** | **Seed shattering** | **G** |
|  | **100916** | **China** | **GU220982*** | **Seed shattering** | **G** |
|  | **106168** | **Vietnam** | **GU221001*** | **Seed shattering** | **G** |
|  | **105319** | **India** | **EU999937*** | **Seed shattering** | **G** |
|  | **81977** | **Indonesia** | **JN679264** | **Seed shattering** | **T** |
|  | **93283** | **Nepal** | **JN679265** | **Seed shattering** | **T** |
|  | **105422** | **Sri Lanka** | **JN679266** | **Seed shattering** | **T** |
|  | **EP18** | **Guangdong, China** | **JN679267** | **Seed shattering** | **T** |
|  | **106452** | **Indonesia** | **JN679268** | **Seed shattering** | **T** |
|  | **103407** | **Sri Lanka** | **EU999934*** | **Seed shattering** | **T** |
|  | **80505** | **India** | **EU999892*** | **Seed shattering** | **G** |
|  | **80534** | **India** | **EU999894*** | **Seed shattering** | **G** |
|  | **80542** | **India** | **EU999895*** | **Seed shattering** | **G** |
|  | **VOC4** | **Nepal** | **EU999927*** | **Seed shattering** | **G** |
|  | **6113** | **Guangxi, China** | **EU999922*** | **Seed shattering** | **G** |
|  | **6803** | **Yunnan, China** | **EU999923*** | **Seed shattering** | **G** |
|  | **105800** | **Thailand** | **JN679269** | **Seed shattering** | **G** |
|  | **TY18** | **Guangdong, China** | **JN679270** | **Seed shattering** | **G** |
|  | **VOC4** | **Nepal** | **EU999926*** | **Seed shattering** | **G** |
|  | **106346** | **Myanmar** | **GU221004*** | **Seed shattering** | **G** |
|  | **103423** | **Sri Lanka** | **EU999898*** | **Seed shattering** | **G** |
|  | **106122** | **India** | **GU220998*** | **Seed shattering** | **G** |
|  | **104311** | **Thailand** | **EU999899*** | **Seed shattering** | **G** |
|  | **104311** | **Thailand** | **EU999900*** | **Seed shattering** | **G** |
|  | **105494** | **Myanmar** | **EU999901*** | **Seed shattering** | **G** |
|  | **105494** | **Myanmar** | **EU999902*** | **Seed shattering** | **G** |
|  | **105720** | **Cambodia** | **EU999903*** | **Seed shattering** | **G** |
|  | **105720** | **Cambodia** | **EU999904*** | **Seed shattering** | **G** |
|  | **105958** | **Indonesia** | **EU999907*** | **Seed shattering** | **G** |
|  | **5213** | **Hainan, China** | **EU999917*** | **Seed shattering** | **G** |
|  | **6109** | **Guangxi, China** | **EU999918*** | **Seed shattering** | **G** |
|  | **6110** | **Guangxi, China** | **EU999920*** | **Seed shattering** | **G** |
|  | **6109** | **Guangxi, China** | **EU999919*** | **Seed shattering** | **G** |
|  | **6113** | **Guangxi, China** | **EU999921*** | **Seed shattering** | **G** |
|  | **Yuan1-10** | **Yunnan, China** | **EU999924*** | **Seed shattering** | **G** |
|  | **Yuan3-9** | **Yunnan, China** | **EU999925*** | **Seed shattering** | **G** |
|  | **93191** | **Nepal** | **EU999930*** | **Seed shattering** | **G** |
|  | **93191** | **Nepal** | **EU999931*** | **Seed shattering** | **G** |
|  | **101967** | **India** | **EU999932*** | **Seed shattering** | **G** |
|  | **103407** | **Sri Lanka** | **EU999933*** | **Seed shattering** | **G** |
|  | **103416** | **Sri Lanka** | **EU999935*** | **Seed shattering** | **G** |
|  | **106345** | **Myanmar** | **EU999947*** | **Seed shattering** | **G** |
|  | **106103** | **India** | **GU220997*** | **Seed shattering** | **G** |
|  | **106134** | **India** | **GU220999*** | **Seed shattering** | **G** |
|  | **105888** | **Bangladesh** | **JN679271** | **Seed shattering** | **G** |
|  | **105902** | **Bangladesh** | **JN679272** | **Seed shattering** | **G** |
|  | **106081** | **India** | **JN679273** | **Seed shattering** | **G** |
|  | **106083** | **India** | **JN679274** | **Seed shattering** | **G** |
|  | **106327** | **Cambodia** | **JN679275** | **Seed shattering** | **G** |
|  | **106328** | **Cambodia** | **JN679276** | **Seed shattering** | **G** |
|  | **Lao16-26** | **Laos** | **JN679277** | **Seed shattering** | **G** |
|  | **105431** | **Sri Lanka** | **EU999939*** | **Seed shattering** | **G** |
|  | **105742** | **Cambodia** | **EU999942*** | **Seed shattering** | **G** |
|  | **106148** | **Laos** | **EU999945*** | **Seed shattering** | **G** |
|  | **106155** | **Laos** | **EU999946*** | **Seed shattering** | **G** |
|  | **105720** | **Cambodia** | **GU220993*** | **Seed shattering** | **G** |
|  | **106150** | **Laos** | **GU221000*** | **Seed shattering** | **G** |
|  | **93277** | **Nepal** | **JN679278** | **Seed shattering** | **G** |
|  | **93278** | **Nepal** | **JN679279** | **Seed shattering** | **G** |
|  | **93280** | **Nepal** | **JN679280** | **Seed shattering** | **G** |
|  | **105420** | **Sri Lanka** | **JN679281** | **Seed shattering** | **G** |
|  | **106075** | **India** | **JN679282** | **Seed shattering** | **G** |
|  | **106080** | **India** | **JN679283** | **Seed shattering** | **G** |
|  | **106152** | **Laos** | **JN679284** | **Seed shattering** | **G** |
|  | **106153** | **Laos** | **JN679285** | **Seed shattering** | **G** |
|  | **106326** | **Cambodia** | **JN679286** | **Seed shattering** | **G** |
|  | **106336** | **Cambodia** | **JN679287** | **Seed shattering** | **G** |
|  | **106391** | **Myanmar** | **JN679288** | **Seed shattering** | **G** |
|  | **105942** | **Thailand** | **EU999905*** | **Seed shattering** | **G** |
|  | **105942** | **Thailand** | **EU999906*** | **Seed shattering** | **G** |
|  | **106453** | **Indonesia** | **EU999911*** | **Seed shattering** | **G** |
|  | **105802** | **Thailand** | **JN679289** | **Seed shattering** | **G** |
|  | **105804** | **Thailand** | **JN679290** | **Seed shattering** | **G** |
|  | **106453** | **Indonesia** | **JN679291** | **Seed shattering** | **G** |
|  | **0112** | **Jiangxi, China** | **EU999913*** | **Seed shattering** | **G** |
|  | **0114** | **Jiangxi, China** | **EU999915*** | **Seed shattering** | **G** |
|  | **80470** | **India** | **EU999928*** | **Seed shattering** | **G** |
|  | **80470** | **India** | **EU999929*** | **Seed shattering** | **G** |
|  | **104871** | **Thailand** | **GU220988*** | **Seed shattering** | **G** |
|  | **80506** | **India** | **EU999893*** | **Seed shattering** | **G** |
|  | **80542** | **India** | **EU999896*** | **Seed shattering** | **G** |
|  | **106453** | **Indonesia** | **EU999912*** | **Seed shattering** | **G** |
|  | **105958** | **Indonesia** | **EU999908*** | **Seed shattering** | **G** |
|  | **105960** | **Bangladesh** | **EU999909*** | **Seed shattering** | **G** |
|  | **106161** | **Laos** | **EU999910*** | **Seed shattering** | **G** |
|  | **106061** | **India** | **EU999944*** | **Seed shattering** | **G** |
|  | **2507** | **Guangdong, China** | **EU999916*** | **Seed shattering** | **G** |
|  | **EP9** | **Guangdong, China** | **JN679292** | **Seed shattering** | **G** |
|  | **0112** | **Jiangxi, China** | **EU999914*** | **Seed shattering** | **G** |
|  | **80774** | **Philippines** | **EU999897*** | **Seed shattering** | **G** |
|  | **106075** | **India** | **JN679293** | **Seed shattering** | **G** |
|  | **106082** | **India** | **JN679294** | **Seed shattering** | **G** |
|  | **104687** | **India** | **EU999936*** | **Seed shattering** | **G** |
|  | **105705** | **Nepal** | **EU999940*** | **Seed shattering** | **G** |
|  | **104599** | **Sri Lanka** | **JN679295** | **Seed shattering** | **G/T** |
|  | **104612** | **Sri Lanka** | **JN679296** | **Seed shattering** | **G/T** |
|  | **104599** | **Sri Lanka** | **GU220984*** | **Seed shattering** | **G/T** |
|  | **105419** | **Sri Lanka** | **JN679297** | **Seed shattering** | **G/T** |
|  | **104624** | **China** | **GU220985*** | **Seed shattering** | **T** |
|  | **105391** | **Thailand** | **EU999938*** | **Seed shattering** | **G** |
|  | **81990** | **Myanmar** | **GU220979*** | **Seed shattering** | **G** |
|  | **105388** | **Thailand** | **GU220989*** | **Seed shattering** | **G** |
|  | **106147** | **Laos** | **JN679298** | **Seed shattering** | **G** |
|  | **106337** | **Myanmar** | **JN679299** | **Seed shattering** | **T** |
|  | **106407** | **Vietnam** | **JN679300** | **Seed shattering** | **T** |
|  | **105491** | **Malasia** | **GU220990*** | **Seed shattering** | **G** |
|  | **105568** | **Philippines** | **GU220991*** | **Seed shattering** | **T** |
|  | **105711** | **India** | **GU220992*** | **Seed shattering** | **G** |
|  | **105855** | **Thailand** | **GU220994*** | **Seed shattering** | **G** |
|  | **106321** | **Cambodia** | **GU221003*** | **Seed shattering** | **G** |
|  | **106518** | **Vietnam** | **GU221006*** | **Seed shattering** | **G** |
|  | **WC8** | **Hainan, China** | **JN679301** | **Seed shattering** | **G** |
|  | **106523** | **Papua New Guinea** | **GU221007*** | **Seed shattering** | **G** |
|  | **105424** | **Sri Lanka** | **JN679302** | **Seed shattering** | **G** |
|  | **105433** | **Sri Lanka** | **JN679303** | **Seed shattering** | **T** |
|  | **106336** | **Myanmar** | **JN679304** | **Seed shattering** | **G** |
|  | **106410** | **Vietnam** | **JN679305** | **Seed shattering** | **G** |
|  | **GZ25** | **Guangdong, China** | **JN679306** | **Seed shattering** | **G** |
|  | **101971** | **India** | **JN679307** | **Seed shattering** | **T** |
|  | **101978** | **India** | **JN679308** | **Seed shattering** | **T** |
