# Supplementary material for: Sequence polymorphisms in wild, weedy, and cultivated rice suggest seed-shattering locus sh4 played a minor role in Asian rice domestication
Source: Ecol Evol. 2012 Jul 24;2(9):2106–13. doi: 10.1002/ece3.318 (PMC3488663; doi:10.1002/ece3.318)
Supplement: Supplementary file 2 [file ece30002-2106-SD2.doc]

| **Appendix 2. Aligned DNA sequences of all the 105 haplotypes of the sh4 locus detected in wild, weedy and cultivated rice** | |
| --- | --- |
| H1 | GTTCTTGATACTTCCATCGCCGTAAACGCCTATTAAAAGACGAGGTTGCGACTCCGAGCCATAGGGTAACAAATTGCTAAACCCTAATATGTTAGCATGGTGT-CGACAATAGAACGGAATAACCTTCCGGGTGCCGGGGCGACGGGGCCGGCGCCGCCGCCGCCGTCGGGTCGTGATACCGGCGGCGCGGCCGCCGCCCCGGCGTGGCGAAACCGG |
| H2 | GTTCTTGATACTTCCATCGCCGTAAACGCCTATTAAAAGACGAGGTTGCGACTCCGAGCCATAGGGTAACAAATTGCTAAACCCTAATATGTTAGCATGGTGT-CGACAATAAAACGGAATAACCTTCCGGGTGCCGGGGCGACGGGGCCGGCGCCGCCGCCGCCGTCGGGTCGTGATACCGGCGGCGCGGCCGCCGCCCCGGCGTGGCGAAACCGG |
| H3 | GTCCTTGATACTTCCGTCGCCGCAAACGC------------GAGGTTGCGGCTGCGAGCCATAGGGTAACAAATTGCTAAACCCTAATATGTTAGCCTGGTGT-CGACAATAGAACGGAATAACCTTGCGGGTGCCGGGGCGACGGGGCCGGCGCCGCCGCCGCCGTCGGGGCGTGATACCGGCGGCGCGGCCGCCGCCCCGGCGTGGCGAGACCGG |
| H4 | GTTCTTGATACTTCCATCGCCGCAAACGCCTATTAAAAGACGAGGTTGCGGCTCCGAGCCATAGGGTAACAAATTGCTAAACCCTAATATGTTAGCATGGTGT-CGACAATAGAACGGAATAACCTTGCGGGTGCCGGGGCGACGGGGCCGGCGCCGCCACCGCCGTCGGGGCGTGATACCGGCGGCGCGGCCGCCGCCCCGGCGCGGCGAAACCGG |
| H5 | GTTCTTGATACTTCCATCGTCGCAAACGCCTATTAAAAGACGAGGTTGCGGCTGCGAGCCATAGGGTAACAAATTGCTAAACCCTAATATGTTAGCATGGTGT-CGACAATAGAACGGAATAACCTTGCGGGTGCCGGGGCGACGGGGCCGGCGCCGCCGCCGCCGTCGGGGCGTGATACCGGCGGCGCGGCCGCCGCCCCGGCGTGGCGAGACCGG |
| H6 | GTCCTTCATACTTCCGTCGCCGCAAACGTCTATTAAAAGACGAGGTTGTGGCTGCGAGC----------------------C--------------CCGGTGT-CGACAATAGAACGAAATAACCTTGCGGGTGCCGGGGCGACGGGGCCTGCGCCGCCGCCGCCGTCGGGGCGTGATACCGGCGGCGCGGCCGCCGCCCCGGCGTGGCGAGACCGG |
| H7 | GTTCTTGATACTTCCGTCGCCGCAAACGCCTATTAGAAGGCGAGATTGCAGCTGCGAGCCACAGGGTAACAAATTGCTAAACCCTAATATGTTAGCCTGGTGT-CGACAATAGAACGGAATAACCTTGCGGGTGCCGGGGCGACGGGGCCGGCGCCGCCGCCGCCGTCGGGGCCTGAT---------------------CCGGCGTGGCGAGACCGG |
| H8 | GTTCTTGATACTTCCGTCGCCGCAAACGCCTATTAAAAGACGAGGTTGCGGCTGCGAGCCATAGGGTAACAAATTGCTAAACCCTAATATGTTAGCCTGGTGT-CGACAATAGAACGGAATAACCTTGCGGGTGCCGGGGCGACGGGGCCGGCGCCGCCGCCGCCGTCGAGGCGTGATACCGGCGGCGCGGCCGCCGCCCCGGCGTGGCGAGACCGG |
| H9 | GTCCTTGATAGTTCCGTCGCCGCAAACGC------------GAGGTTGCGGCTGCGAGCCATAGGGTAACAAATTGCTAAACCCTAATATGTTAGCCTGGTGT-CGACAATAGAACGGAATAACCTTGCGGGTGACGGGGCGACGGGGCCGGCGCCGCCGCCGCCGTCGGGGCGTGAT------------------GCCCCGGCGTGGCGAGACCGG |
| H10 | GTTCTTGATACTTCCATCGCCGCAAACGCCTATTAAAAGACGAGGTTGCGACTCCGAGCCATAGGGTAACAAATTGCTAAACCCTAATATGTTAGCATGGTGT-CGACAATAGAACGGAATAACCTTCCGGGTGCCGGGGCGACGGGGCCGGCGCCGCCGCCGCCGTCGGGTCGTGATACCGGCGGCGCGGCCGCCGCCCCGGCGTGGCGAGACCGG |
| H11 | GTTCTTGATACTTCCGTCGCCGCAAACGCCTATTAAAAGACGAGGTTGCGACTGCGAGCCATAGGGTAACAAATTGCTAAACCCTAATATGTTAGCATGGTGT-CGACAATAGAACGGAATAACCTTCCGGGTGCCGGGGCGACGGGGCCGGCGCCGCCGCCGCCGTCGGGTCGTGATACCGGCGGCGCGGCCGCCGCCCCGGCGTGGCGAAACCGG |
| H12 | GTTCTTGATACTTCCATCGCCGCAAACGCCTATTAAAAGACGAGGTTGCGGCTCCGAGCCATAGGGTAACAAATTGCTAAACCCTAATATGTTAGCATGGTGT-CGACAATAGAACGGAATAACCTTCCGGGTGCCGGGGCGACGGGGCCGGCGCCGCCGCCGCCGTCGGGTCGTGATACCGGCGGCGCGGCCGCCGCCCCGGCGTGGCGAAACCGG |
| H13 | GTTCTTGATACTTCCATCGTCGTAAACGCCTATTAAAAGACGAGGTTGCGGCTCCGAGCCATAGGGTAACAAATTGCTAAACCCTAATATGTTAGCATGGTGT-CGACAATAGAACGGAATAACCTTCCGGGTGCCGGGGCGACGGGGCCGGCGCCGCCGCCGCCGTCGGGTCGTGATACCGGCGGCGCGGCCGCCGCCCCGGCGTGGCGAAACCGG |
| H14 | GTTCTTGATACTTCCGTCGCCGTAAACGCCTATTAAAAGACGAGGTTGCGACTCCGAGCCATAGGGTAACAAATTGCTAAACCCTAATATGTTAGCATGGTGT-CGACAATAGAACGGAATAACCTTCCGGGTGCCGGGGCGACGGGGCCGGCGCCGCCGCCGCCGTCGGGTCGTGATACCGGCGGCGCGGCCGCCGCCCCGGCGTGGCGAAACCGG |
| H15 | GCTCTTGATACTTCCATCGCCGTAAACGCCTATTAAAAGACGAGGTTGCGACTCCGAGCCATAGGGTAACAAATTGCTAAACCCTAATATGTTAGCATGGTGT-CGACAATAGAACGGAATAACCTTCCGGGTGCCGGGGCGACGGGGCCGGCGCCGCCGCCGCCGTCGGGTCGTGATACCGGCGGCGCGGCCGCCGCCCCGGCGTGGCGAAACCGG |
| H16 | GTTCTTGATACTTTCGTCGCCGCAAACGCCTATTAAAAGACGAGGTTGCGGCTGCGAGCCATAGGGTAACAAATTGCTAAACCCTAATATGTTAGCATGGTGT-ATACAATAGAACGGAATAACCTTGCGGGTGCCGGGGCGACGGGGCCGGCGCCGCCGCCGCCGTCGGGGCGTGAT------------------GCCCCGGCGTGGCGAGACCGG |
| H17 | GTTCTTGATACTTCCATCGCCGCAAACGCCTATTAAAAGACGAGGTTGCGGCTCCGAGCCATAGGGTAGCAAATTGCTAAACCCTAATATGTTAGCATGGTGT-CGACAATAGAACGGAATAACCTTGCGGGTGCCGGGGCGACGGGGCCGGCGCCGCCGCCGCCGTCGGGGCGTGATACCGGCGGCGCGGCCGCCGCCCCGGCGTGGCGAAACCGG |
| H18 | GTTCTTGATACTTCCATCGCCGCAAACGCCTATTAAAAGACGAGGTTGCGGCTCCGAGCCATAGGGTAACAAATTGCTAAACCCTAATATGTTAGCATGGTGT-CGACAATAGAACGGAATAACCTTGCGGGTGCCGGGGCGACGGGGCCGGCGCCGCCGCCGCCGTCGGGGCGTGATACCGGCGGCGCGGCCGCCGCCCCGGCGTGGCGAGACCGG |
| H19 | GTTCTTGATACTACCATCGCCGCAAACGCCTATTAAAAGACGAGGTTGCGGCTCCGAGCCATAGGGTAACAAATTGCTAAACCCTAATATGTTAGCATGGTGT-CGACAATAGAACGGAATAACCTTGCGGGTGCCGGGGCGACGGGGCCGGCGCCGCCGCCGCCGTCGGGGCGTGATACCGGCGGCGCGGCCGCCGCCCCGGCGTGGCGAAACCGG |
| H20 | GTTCTAGATACTTCCATCGCCGCAAACGCCTATTAAAAGACGAGGTTGCGGCTCCGAGCCATAGGGTAACAAATTGCTAAACCCTAATATGTTAGCATGGTGT-CGACAATAGAACGGAATAACCTTGCGGGTGCCGGGGCGACGGGGCCGGCGCCGCCGCCGCCGTCGGGGCGTGATACCGGCGGCGCGGCCGCCGCCCCGGCGTGGCGAAACCGG |
| H21 | GTCCTTGATAGTTCCATCGCCGCAAACGCCTATTAGAAGACGAGGTTGCGGCTCCGAGCCATAGGGTAACAAATTGCTAAACCCTAATATGTTAGCCTGGTGT-CGACAATAGAACGGAATAACCTTGCGGGTGCCGGGGCGACGGGGCCGGCGCCGCCGCCGCCGTCGGGGCGTGATACCGGCGGCGCGGCCGCCGCCCCGGCGTGGCGAGACCGG |
| H22 | GTTCTTGATACTTCCATCGCCGCAAACGCCTATTAAAAGACGAGGTTGCGGCTCCGAGCCATAGGGTAACAAATTGCTAAACCCTAATATGTTAGCATGGTGT-CGACAATAGAACGGAATAACCTTGCGGGTGCCGGGGCGACGGGGCCGGCGCCGCCGCCGCCGTCGGGGCGTGATACCGGCGGCGCGGCCGCCGCCCCGGCGTGGCGAAACCGG |
| H23 | GTCCTTCATACTTCCGTCGCCGCAAACACCTATTAAAAGACGAGATTGCGGCTGCGAGCCACAGGGTAACAAATTGCTAAACCCTAATATGTTAGCATGGTGT-CGACAATAGAACGGAATAACCTTGCGGGTGCCGGGGCGACGGGGCCGGCGCCGCCGCCGCCGTCGGGGCGTGATACCGGCGGCGCGGCCGCCGCCCCGGCGTGGCGAGACCGG |
| H24 | GTTCTTGATACTTCCATCGCCGCAAACACCTATTAAAAGACGAGATTGCGGCTGCGAGCCACAGGGTAACAAATTGCTAAACCCTAATATGTTAGCCTGGTGT-CGACAATAGAACGGAATAACCTTGCGGGTGCCGGGGCGACGGGGCCGGCGCCGCCGCCGCCGTCGGGGCGTGATACCGGCGGCGCGGCCGCCGCCCCGGCGTGGCGAGACCGG |
| H25 | GTCCTTGATACTTCCGTCGCCGCAAACGCCTATTAGAAGGCGAGATTGCAGCTGCGAGCCACAGGGTAACAAATTGCTAAACCCTAATATGTTAGCCTGGTGT-CGACAATAGAACGGAATAACCTTGCGGGTGCCGGGGCGACGGGGCCGGCGCCGCCGCCGCCGTCGGGGCGTGATACCGGCGGCGTGGCCGCCGCCCCGGCGTGGCGAGACCGG |
| H26 | GTTCTTGATACTTCCATCGCCGCAAACGCCTATTAGAAGACGAGATTGCAGCTGCGAGCCACAGGGTAACAAATTGCTAAATCCTAATATGTTAGCCTGGTGT-CGACAATAGAACGGAATAACCTTGCGGGTGCCGGGGCGACGGGGCCGGCGCCGCCGCCGCCGTCGGGGCGTGAT------------------GCCCCGGCGTGGCGAGACCGG |
| H27 | GTTCTTGATACTTCCGTCGCCGCAAACGCCTATTAGAAGACGAGATTGCAGCTGCGAGCCACAGGGTAACAAATTGCTAAATCCTAATATGTTAGCCTGGTGT-CGACAATAAAACGGAACAACCTTGCGGGTGCCGGGGCGACGGGGCCGGCGCCGCCGCCGCCGTCGGGGCGTGAT------------------GCCCCGGCGTGGCGAGACCGG |
| H28 | GTTCCTGATACTTCCGCTGCCACAAACGCCTATTAGAAGACGAGATTGCAGCCGCGAGCCACAGGGTAACAAATTGCTAAACCCTAATATGTTAGCCTGGTGT-CGACAGTAGAACGGAATAAACTTGCGGGTGCCGGGGCGACGGGGCCGGCGCCGCCGCCGCCGTCGGGGCGTGAT------------------GCCCCGGTGTGGCGAGACCGG |
| H29 | GTTCTTGATACTTCCGCTGCCACAAACGCCTATTAGAAAACGAGATTGCAGCCGCGAGCCACAGGGTAACAAATTGCTAAACCCTAATATGTTAGCCTGGTGT-CGACAGTAGAACGGAATAAACTTGCGGGTGCCGGGGCGACGGGGCCGGCGCCGCCGCCGCCGTCGGGGCGTGAT------------------GCCCCGGTGTGGCGAGACCGG |
| H30 | GTTCTTGATACTTCCGTCGCCATAAACACTTATTAGAAGACGAGATTGCAGCGGCGAGCCACAGGGTAACAAATTGCTAAACCCTAATATGTTAGCCTGGTGT-CGACAATAAAACGGAATAACCTTGCGGGTGCCGGGGCGACGGGGCCGGCGCCGCCGCCGCCGTCGGGGCGTGAT------------------GCCCCGGCGTGGCGAGACCGG |
| H31 | GTTCTTGATACTTCCGTCGCCATAAACACTTATTAGAGGACGAGATTGCAGCGGCGAGCCACAGGGTAACAAATTGCTAAACCCTAATATGTTAGCCTGGTGT-CGACAATAAAACGGAATAACCTTGCGGGTGCCGGGGCGACGGGGCCGGCGCCGCCGCCGCCGTCGGGGCGTGAT------------------GCCCCGGCGTGGCGGAACCGG |
| H32 | GTTCTTGATACTTCCGTCGCCGCAAACGCCTATTAAAAGACGAGGTTGCGGCTGCGAGCCATAGGGTAACAAATTGCTAAACCCTAATATGTTAGCCTGGTGT-CGACAATAGAACGGAATAACCTTGCGGGTGCCGGGGCGACGGGGCCTGCGCCGCCGCCGCCGTCGGGGCGTGATACCGGCGGCGCGGCCGCCGCCCTGGCGTGGCGAGACCGG |
| H33 | GTCCTAGATACTTCCGTCGCCGCAAACGCCTATTAAAAGACGAGGTTGCGACTGCGAGCCATAGGGTAACAAATTGCTAAACCCTAATATGTTAGCCTGGTGT-CGACAATAGAACGGAATGACCTTGCGGGTGCCGGGGCGACGGGGCCTGCGCCGCCGCCGCCGTCGGGGCGTGATACCGGCGGCGCGGCCGCCGCCCCGGCGTGGCGAGACCGG |
| H34 | GTTCTTGATACCTCCATCGCCGCAAACGCCTATTAAAAGACGAGGTTACGGCTACGAGCCATAGGGTAACAAATTGCTAAACCCTAATATGTTAGCCTGGTGT-CGACAATAGAAAGGAATAACCTTGCGGGTGCCGGGGCGACGGGGCCTGCGCCGCCGCCGCCGTCGGGGCGTGATACCGGCGGCGCGGCCGCCGCCCCGGCGTGGCGAGACCGG |
| H35 | GTTCTTGATACCTCCATCGCCGCAAACGCCTATTAAAAGACGAGGTTACGGCTACGAGCCATAGGGTAACAAATTGCTAAACCCTAATATGTTAGCCTGGTGT-CGACAATAGAACGGAATAACCTTGCGGGTGCCGGGGCGACGGGGCCTGCGCCGCCGCCGCCGTCGGGGCGTGATACCGGCGGCGCGGCCGCCGCCCCGGCGTGGCGAGACCGG |
| H36 | GTTCTTGATACCTCCATCGCCGCAAACGCCTATTAAAAGACGAGGTTACGGCTACGAGCCATAGGGTAACAAATTGCTAAACCCTAATATGTTAGCCTGGTGT-CGACAATAGAAAGGAGTAACCTTGCGGGTGCCGGGGCGACGGGGCCTGCGCCGCCGCCGCCGTCGGGGCGTGATACCGGCGGCGCGGCCGCCGCCCCGGCGTGGCGAGACCGG |
| H37 | GTTCTTGATACTTCCATCGTCGCAAACGCCTATTAAAAGACGGGGTTGCGGCTGCGAGCCATAGGGTAACAAATTGCTAAACCCTAATATGTTAGCATGGTGT-CGACAATAGAACGGAATAACCTTGCGGGTGCCGGGGCGACGGGGCCGGCGCCGCCGCCGCCGTCGGGGCGTGATACCGGCGGCGCGGCCGCCGCCCCGGCGCGGCGAAACCGG |
| H38 | GTTCTTGATACTTCCGTCGCCGCAAACGCCTATTAAAAGACGAGGTTGCGGCTGCGAGCCATAGGGTAACAAATTGCTAAACCCTAATATGTTAGCCTGCTGT-CGACAATAGAACGGAATAACCTTGCGGGTGCCGGGGCGACGGGGCCGGCGCCGCCGCCGCCGTCGGGGCGTGATACCGGCGGCGCGGCCGCCGCCCCGGCGTGGCGAAACCGG |
| H39 | GTTCTTGATACTTCCGTCGCCGCAAACGCCTATTAAAAGACGAGGTTGCGGCTGCGAGCCATAGGGTAACAAATTGCTAAACCCTAATATGTTAGCCTGCTGT-CGACAATAGAACGGAATAACCTTGCGGGTGCCGGGGCGACGGGGCCGGCGCCGCCGCCGCCGTCGGGGCGTGATACCGGCGGCGCGGCCGCCGCCCCGGCGTGGCGAGACCGG |
| H40 | GTTCTTGATACTTCCGTCGCCGCAAACGCCTATTAGAAGACGAGATTGCAGCTGCGAGCCACAGGGTAACAAATTGCTAAATCCTAATATGTTAGCCTGGTGT-CGACAATAGAACGGAATAACCTTGCGGGTGCCGGGGCGACGGGGCCGGCGCCGCCGCCGCCGTCGGGGCGTGAT------------------GCCCCGGCGTGGCGAGACCGG |
| H41 | GCTCTTGATACTTCCGCTGCCACAAACGCCTATTAGAAAACGAGATTGCAGCCGCGAGCCACAGGGTAACAAATTGCTAAACCCTAATATGTTAGCCTGGTGT-CGACAGTAGAACGGAATAAACTTGCGGGTGCCGGGGCGACGGGGCCGGCGCCGCCGCCGCCGTCGGGGCGTGAT------------------GCCCCGGCGTGGCGAGACCGG |
| H42 | GTTCTTGATACTTCCGTCGCCGCAAACGCCTATTAAAAGACGAGGTTGCGGCTGCGAGCCATAGGGTAACAAATTGCTAAACCCTAATATGTTAGCCTGGTGT-CGACAATAGAACGGAATAACCTTGCGGGTGCCGGGGCGACGGGGCCGGCGCCGCCGCCGCCGTCGGGGCGTGATACCGGCGGCGCGGCCGCCGCCCCGGCGTGGCGAGACCGG |
| H43 | GTTCTTGATACTTCCGTCGCCGCAAACGCCTATTAAAAGACGAGGTTGCGGCTGCGAGCCACAGGGTAACAAATTGCTAAACCCTAATATGTTAGCCTGGTGT-CGACAATAGAACGGAATAACCTTGCGGGTGCCGGGGCGACGGGGCCGGCGCCGCCGCCGCCGTCGGGGCGTGATACCGGCGGCGCGGCCGCCGCCCCGGCGTGGCGAGACCGG |
| H44 | GTCCTTGATAGTTCCGTCGCCGCAAACGC------------GAGGTTGCGGCTGCGAGCCATAGGGTAACAAATTGCTAAACCCTAATATGTTAGCCTGGTGT-CGACAATAGAACGGAATAACCTTGCGGGTGCCGGGGCGACGGGGCCGGCGCCGCCGCCGCCGTCGGGGCGTGATACCGGCGGCGCGGCCGCCGCCCCGGCGTGGCGAGACCGG |
| H45 | GTTCTTGATAGTTCCGTCGCCGCAAACGCCTATTAAAAGACGAGGTTGCGGCTGCGAGCCACAGGGTAACAAATTGCTAAACCCTAATATGTTAGCCTGGTGT-CGACAATAGAACGGAATAACCTTGCGGGTGCCGGGGCGACGGGGCCGGCGCCGCCGCCGCCGTCGGGGCGTGATACCGGCGGCGCGGCCGCCGCCCCGGCGTGGCGAGACCGG |
| H46 | GTTCTTGATACTTCCATCGCCGCAAACGCCTATTAAAAGACGAGGTTGCGGCTGCGAGCCATAGGGTAACAAATTGCTAAACCCTAATATGTTAGCCTGGTGT-CGACAATAGAACGGAATAACCTTGCGGGTGCCGGGGCGACGGGGCCGGCGCCGCCGCCGCCGTCGGGGCGTGATACCGGCGGCGCGGCCGCCGCCCCGGCGTGGCGAAACCGG |
| H47 | GTTCTTGATACTTCTGTCGCCGCAAACGCCTATTAAAAGACGAGGTTGCGGCTGCGAGCCATAGGGTAACAAATTGCTAAACCCTAATATGTTAGCCTGGTGT-CGACAATAGAACGGAATAACCTTGCGGGTGCCGGGGCGACGGGGCCGGCGCCGCCGCCGCCGTCGGGGCGTGATACCGGCGGCGCGGCCGCCGCCCCGGCGTGGCGAGACCGG |
| H48 | GTTGTTGATACTTCCGTCGCCGCAAACGCCTATTAAAAGACGAGGTTGCGGCTGCGAGCCATAGGGTAACAAATTGCTAAACCCTAATATGTTAGCATGGTGT-CGACAATAGAACGGAATAACCTTGCGGGTGCCGGGGCGACGGGGCCGGCGCCGCCGCCGCCGTCGGGGCGTGATACCGGCGGCGCGGCCGCCGCCCCGGCGTGGCGAGACCGG |
| H49 | GTTCTTGAAACTTCCGTCGCCGCAAACGCCTATTAAAAGACGAGGTTGCGGCTGCGAGCCATAGGGTAACAAATTGCTAAACCCTAATATGTTAGCCTGGTGT-CGCCAATAGAACGGAATACCCTTGCGGGTGCCGGGGCGACGGGGCCGGCGCCGCCGCCGCCGTCGGGGCGTGATACCGGCGGCGCGGCCGCCGCCCCGGCGTGGCGAGACCGG |
| H50 | GTTCTTGATACTTCCGCTGCCACAAACGCCTATTAGAAAACGAGATTGCCGCCGCGAGCCACAGGGTAACAAATTGCTAAACCCTAATATGTTAGCCTGGTGT-CGACAGTAGAACGGAATAAACTTGCGGGTGCCGGGGCGACGGGGCCGGCGCCGCCGCCGCCGTCGGGGCGTGAT------------------GCCCCGGCGTGGCGAAACCGG |
| H51 | GTTCTTGATACTTCCGTCGCCGCAAACGCCTATTAAAAGACGAGGTTGCGGCTGCGAGCCATAGGGTAACAAATTGCTAAACCCTAATATGTTAGCCTGGTGT-CGCCAATAGAACGGAATACCCTTGCGGGTGCCGGGGCGACGGGGCCGGCGCCGCCGCCGCCGTCGGGGCGTGAT------------------GCCCCGGCGTTGCGAGACCGG |
| H52 | GTCCTTGATACTTCCATCGCCACCAACGCCTATTAAAAGACGAGGCTGCGGCTGCAGGCCATAGGGTAACAAATTGCTAAACCCTAATATGTTAGCATGGTGT-CGACAATAAAACGGAATAACCTTGCGGGTGCCGGGGCGACGGGGCCGGCGCCGCCGCCGCCGTCGGGGCGTGATACCGGCGGCGCGGCCGCCGCCCCGGCGTGGCAAGACCGG |
| H53 | GTCCTTGATAGTTCCGTCGTCGCAAACGCCTAT--------GAGGTTGCGGCTGCGAGCCACAGGGTAACAAATTGCTAAACCCTAATATGTTAGCCTGGTGT-CGACAATAGAACGGAATAACCTTGCGGGTGCCGGGGCGACGGGGCCGGCGCCGCCGCCGCCGTCGGGGCGTGATACCGGCGGCGCGGCCGCCGCCCCGGCGTGGCGAGACCGG |
| H54 | GTTCTTGATACTTCCATCGCCGCAAACACCTATTAAAAGACGAGGTTGTGGCTGCGAGCCACAGGGTAACAAATTGCTAAACCCTAATATGTTAGCATGGTGT-CGACAATAGAACGGAATAACCTTGCGGGTGCCGGGGCGACGGGGCCGGCGCCGCCGCCGCCGTCGGGGCGTGATACCGGCGGCGCGGCCGCCGCCCCGGCGTGGCGAGACCGG |
| H55 | GTCCTTGATACTTCCGTCGCCGCAAACGCCTATTAAAAGGCGAGATTGCGGCTGCGAGCCACAGGGTAACAAATTGCTAAACCCTAATATGTTAGCCTGGTGT-CGACAATAGAACGGAATAACCTTGCGGGTGCCGGGGCGACGGGGCCGGCGCCGCCGCCGCCGTCGGGGCGTGATACCGGCGGCGCGGCCGCCGCCCCGGCGTGGCGAGACCGG |
| H56 | GTTCTTGATACTTCCATCGCCGCAAACGCCTATTAAAAGACGAGGTTGCGGCTGCGAGCCATAGGGTAACAAATTGCTAAACCCTAATATGTTAGCCTGGTGT-CGACAATAGAACGGAATAACCTTGCGGGTGCCGGGGCGACGGGGCCGGCGCCGCCGCCGCCGTCGGGGCGTGATACCGGCGGCGCGGCCGCCGCCCCGGCGCGGCGAAACCGG |
| H57 | GTCCTTGATACTTCCGTCGCCGCAAACGCCTATTAAAAGACGAGGTTGCGACTGCGAGCCATAGGGTAACAAATTGCTAAACCCTAATATGTTAGCCTGGTGT-CGACAATAGAACGGAATAACCTTGCGGGTGCCGGGGCGACGGGGCCGGCGCCGCCGCCGCCGTCGGGGCGTGATACCGGCGGCGCGGCCGCCGCCCCGGCGTGGCGAGACCGG |
| H58 | GTTCTTGATAGTTCCGTCGCCGCAAACGCCTATTAGAAGACAAGATTGCAGCTGCGAGCCACAGGGTAACAAATTGCTAAACCCTAATATGTTAGCCTGGTGT-CGACAATAGAACGGGATAACCTTGCGGGTGCCGGGGCGACGGGGCCGGCGCCGCCGCCGCCGTCGGGGCGTGATACCGGCGGCGCGGCCGCCGCCCCGGCGTGGCGAGACCGG |
| H59 | GTCCTTGATACTTCCGTCGCCGCAAATGC------------GAGGTTGCGGCTGCGAGCCATAGGGTAACAAATTGCTAAACCCTAATATGTTAGCCTGGTGC-CGACAATAGAACGGAATAACCTTGCGGGTGCCGGGGCGACGGGGCCGGCGCCGCCGCCGCCGTCGGGGCGTGAT------------------GCCCCGGCGTGGCGAGACCGG |
| H60 | GTCCTTGATACTTCCGTCGCCGCAAGCGCCTATTAAAAGACGAGGTAGCGGCTGCGAACCACAGGGTAACAAATTGCTAAACCCTAATATGTTAGCCTGGTGT-CGACAATAGAACGGAATAACCTTGCGGGTGCCGGGGCGACGGGGCCTGCGCCGCCGCCGCCGTCGGGGCGTGATACCGGCGGCGCGGCCGCCGCCCTGGCGTGGCGAGACCGG |
| H61 | TTCCTTGATACTTCCGTCACCGCAAACACCTATTAGAAGACGAGATTGCAGCTGCGAGCCACAGGGTAACAAATTGCTAAACCCTAATATGTTAGCCTGGTGT-CGACAATAGAACGGAATAACCTTGCGGGTGCCGGGGCGACGGGGCCGGCGCCGCCGCCGCCGTCGGGGCGTGAT------------------GCCCCGGCATGGCGAGACCGG |
| H62 | GTCCTTGATACTTCCGTCGCCGCAAACGCCTATTAGAAGGCGAGATTGCAGCTGCGAGCCACAGGGTAACAAATTGCTAAACCCTAATATGTTAGCCTGGTGT-CGACAATAGAACGGAATAACCTTGCGGGTGCCGGGGCGACGGGGCCGGCGCCGCCGCCGCCGTCGGGGTGTGAT------------------GCCCCGGCGTGGCGAGACCGG |
| H63 | GTTGTTGATACTTCCGTCGCCGCAAACACCTATTAAAAGACGAGATTGCGGCTGCGAGCCACAGGGTAACAAATTGCTAAACCCTAATATGTTAGCCTGGTGT-CGACAATAGAACGGAATAACCTTGCGGGTGCCGGGGCGACGGGGCCGGCGCCGCCGCCGCCGTCGGGGTGTGATACCGGCGGCGCGGCCGCCGCCCCGGCGTGGCGAGACCGG |
| H64 | GTTCTTGATACTTCCGTCGCCGCAAACGCCTATTAAAAGACGAGGTTGCAGCTGCGAGCCACAGGGTAACAAATTGCTAAACCCTAATATGTTAGCCTGCTGT-CGACAATAGAACGGAGTAACCTTGCGGGTGCCGGGGCGACGGGGCCGGCGCCGCCGCCGCCGTCGGGGCCTGAT---------------------CCGGCGTGGCGAGACCGG |
| H65 | GTTCTTCATACTTCCGTCGCCGCAAACACCTATTAAAAGACGAGATTGCGGCTGCGAGCCACAGGGTAACAAATTGCTAAACCCTAATATGTTAGCCTGGTGT-CGACAATAGAACGGAATAACCTTGCGGGTGCCGGGGCGACGGGGCCGGCGCCGCCGCCGCCGTCGGGGCCTGATACCGGCGGCGCGGCCGCCGCCCCGGCGTGGCGAGACCGG |
| H66 | GTTCTTGATACTTCCGTCGCCGCAAACGCCTATTAGAAGACGAGATTGCAGCTGCGAGCCACAGGGTAACAAATTGCTAAACCCTAATATGTTAGCCTGGTGT-CGACAATAGAACGGAATAACCTTGCGGGTGCCGGGGCGACGGGGCCGGCGCCGCCGCCGCCGTCGGGGCCTGAT---------------------CCGGCGTGGCGAGACCGG |
| H67 | GTTCTTGATACTTCCATCGTCGCAAACGCCTATTAAAAGACGAGGTTGCGGCTGCGAGCCATAGGGTAACAAATTGCTAAACCCTAATATGTTAGCCTGGTGT-CGACAATAGAACGGAATAACCTTGCGGGTGCC---------------------------------GGGCGTTATACCGGCGGCGCGGCCGCCGCCCCGGCGTGGCGAAACCGG |
| H68 | GTTCTTGATACTTCCGTCGCTGCAAACGCCTATTAGAAGACGAGATTGCAGTTCGGAGCCACAGGGTAACAAATTGCTAAACCCTAATATGTTAGCCTGGTGT-CGACAATAGAACGGAATAACCTTGCGGGTGCCGGGGCGACGGGGCCGGCGCCGCCGCCGCCGTCGGGGCGTGAC------------------GCCCCGGCGTGGCGAGACCGG |
| H69 | GTTCTTGATACTTCCGTCGCTGCAAACGCCTATTAGAAGACGAGATTGCAGTTGCGAGCCACAGGGTAACAAATTGCTAAACCCTAATATGTTAGCCTGGTGT-CGACAATAGAACGGAATAACCTTGCGGGTGCCGGGGCGACGGGGCCGGCGCCGCCGCCGCCGTCGGGGCGTGAC------------------GCCCCGGCGTGGCGAAACCGG |
| H70 | GTTCTTGATACTTCCGTCGCCGCAAACGCCTATTAAAAGACGAGGTTGCGACTCCGAGCCATAGGGTAACAAATTGCTAAACCCTAATATGTTAGCCTGGTGT-CGACAATAGAACGGAATAACCTTGCGGGTGCCGGGGCGACGGGGCCGGCGCCGCCGCCGCCGTCGGGGCGTGAT------------------GCCTCGGCGTGGCGAGACCGG |
| H71 | GTCCTTGATACTTCCGTCGCCGCAAACGCCTATTAAAAGACGAGGTTGCGGCTGCGAGCCACAGGGTAACAAATTGCTAAACCCTAATATGTTAGCATGGTGT-CGAGAATAGAACGGAATAACCTTGCGGGTGCCGGGGCGACGGGGCCTGCGCCGCCGCCGCCGTCGGGGCGTGATACCGGCGGCGCGGCCGCCGCCCCGGCGTGGCGAGACTGG |
| H72 | GTCCTTGATACTTCCGTCGCCGCAAACACCTATTAAAAGACGAGATTGCGGCTGCGAGCCACAGGGTAACAAATTGCTAAACCCTAATATGTTAGCATGGTGT-CGACAATAGAACGGAATAACCCTGCGGGTGCCGGGGCGACGGGGCCTGCGCCGCCGCCGCCGTCGGGGCGTGATACCGGCGGCGTGGGGGGGGCCCCGGCGTGGCGAGACTGG |
| H73 | GTTCTTGATACTTCCGTCGCCGCAAACGCCTATTAAAAGACGAGGTTGCGGCTGCGAGCCATAGGGTAACAAATTGCTAAACCCTAATATGTTAGCCTGCTGT-CGACAATTAAACGGAGTAACCTTGCGGGTGCCGGGGCGACGGGGCCGGCGCCGCCGCCGCCGTCGGGGCCTGAT---------------------CCGGCGTGGCGAGACTGG |
| H74 | GTTCTTGATACTTCCGTCGCCGCAAACGCCTATTAAAAGACGAGGTTGCGGCTGCGAGCCATAGGGTAACAAATTGCTAAACCCTAATATGTTAGCCTGGTGT-CGACAATAGAACGGAATACCCTTGCGGGTGCCGGGGCGACGGGGCCGGCGCCGCCGCCGCCGTCGGGGCGTGAT------------------GCCCCGGCGTGGCGAGACCGG |
| H75 | GTCCTTGATACTTCCGTCGCCGCAAACGCCTATTAGAAGGCGAGATTGCAGCTGCGAGCCACAGGGTAACAAATTGCTAAACCCTAATATGTTAGCCTGGTGT-CGACAATAGAACGGAATAACCTTGAGGGTGCCGGGGCGACGGGGCCGGCGCCGCCGCCGCCGTCGGGGCGTGGT------------------GCCCCGGCGTGGCGAGACCGG |
| H76 | GTTCTTGATACTTCCATCGCCGTAAACGCCTATTAAAAGACGAGGTTGCGACTCCGAGCCATAGGGTAACAAATTGCTAAACCCTAATATGTTAGCATGGTGT-CGACAATAGAACGGAATAACCTTCCGGGTGCCGGGGCGACGGGGCCGGCGCCGCCGCCGCCGTCGGGGCGTGATACCGGCGGCGCGGCCGCCGCCCCGGCGTGGCGAGACCGG |
| H77 | GTTCTTGATACTTCCATCGCCGCAAACGCCTATTAAAAGACGAGGTTGCGGCTGCGAGCCATAGGGTAACAAATTGCTAAACCCTAATATGTTAGCATGGTGT-CGACAATAGAACGGAATAACCTTCCGGGTGCCGGGGCGACGGGGCCGGCGCCGCCGCCGCCGTCGGGGCGTGATACCGGCGGCGCGGCCGCCGCCCCGGCGTGGCGAAACCGG |
| H78 | GTTCTTGATACTTCCATCGCCGCAAACACCTATTAAAAGACGAGGTTGCGGCTGCGAGCCATAGGGTAACAAATTGCTAAACCCTAATATGTTAGCATGGTGT-CGACAATAGAACGGAATAACCTTCCGGGTGCCGGGGCGACGGGGCCGGCGCCGCCGCCGCCGTCGGGGCGTGATACCGGCGGCGCGGCCGCCGCCCCGGCGTGGCGAAACCGG |
| H79 | GTTCTTGATACTTCCATCGTCGCAAACGCCTATTAAAAGACGAGGTTGCGGCTGCGAGCCATAGGGTAACAAATTGCTAAACCCTAATATGTTAGCCTGGTGT-CGACAATAGAACGGAATAAGTTTGCGGGTGCC---------------------------------GGGCGTTATACCGGCGGCGCGGCCGCCGCCCCGGCGTGGCGAAACCGG |
| H80 | GTTCTTGATACTTCCATCGCCGCAAACGCCTATTAAAAGACGAGGTTGCGGCTCCGAGCCATAGGGTAACAAATTGCTAAACCCTAATATGTTAGCATGGTGT-CGACAATAGAACGGAATAACCTGGCGGGAGCCGGGGCGACGGGGCCGGCGCCGCCGCCGCCGTCGGGGCGTGATACCGGCGGCGCGGCCGCCGCCCCGGCGTGGCGAGACCGG |
| H81 | GTCCTTGATACTTCCATCGCCGCAAACGCCTATTAAAAGACGAGGTTGCGGCTCCGAGCCATAGGGTAACAAATTGCTAAACCCTAATATGTTAGCATGGTGT-CGACAATAGAACGGAATAACCTGGCGGGAGCCGGGGCGACGGGGCCGGCGCCGCCGCCGCCGTCGGGGCGTGATACCGGCGGCGCGGCCGCCGCCCCGGCGTGGCGAGACCGG |
| H82 | GTTCTTGATACTTCCGTCGCCGCAAACGCCTATTAAAAGACGAGGTTGCGGCTGCGAGCCATAGGGTAACAAATTGCTAAACCCTAATATGTTAGCCTGCTGT-CGACAATAGAACGGAATAACCTTGCGGGTGCCGGGGCGACGGGGCCGGCGCCGCCGCCGCCGTCGGGGCGTGATACCGGCGGCGCGGCCGCCGCCCCGGCGTGGCGAGACCAG |
| H83 | GTTCTTGATACTTCCGTCGCCGCAAACGCCTATTAAAAGACGAGGTTGCGGCTGCGAGCCATAGGGTAACAAATTGCTAAACCCTAATATGTTAGCCTGCTGT-CGACAATAGAACAGAATAACCTTGCGGGTGCCGGGGCGACGGGGCCGGCGCCGCCGCCGCCGTCGGGGCGTGATACCGGCGGCGCGGCCGCCGCCCCGGCGTGGCGAGACCAG |
| H84 | GTTCTTGATACTTCCATCGCCGCAAACGCCTATTAAAAGACGAGGTTGCAGCTCCGAGCCACAGGGTAACAAATTGCTAAACCCTAATATGTTAGCCTGGTGT-CGACAATAGAACGGAATAACCTTCCGGGTGCCGGGGCGACGGGGCCGGCGCCGCCGCCGCCGTCGGGTCGTGATACCGGCGGCGCGGCCGCCGCCCCGGCGTGGCGAAACCGG |
| H85 | GTTCTTGATACTTCCGCTGCCACACACGCCTATTAGAAAACGAGATTGCAGCCGCGAGCCACAGGGTAACAAATTGCTAAACCCTAATATGTTAGCCTGGTGT-CGACAGTAGAACGGAATAAACTTGCGGATGCCGGGGCGACGGGGCCGGCGCCGCCGCCGCCGTCGGGGCGTGAT------------------GCCCCGGCGTGGCGAGACCGG |
| H86 | GTTCTTGATACTTCCGTCGCCGCAAACGCCTATTAAAAGACGAGGTTGCGGCTGCGAGCCATAGGGTAACAAATTGCTAAACCCTAATATGTTAGCCTGGTGT-CGACAATAGAACGGAATAACCTTGCGGGTGCCGGGGCGACGGGGCCGGCGCCGCCGCCGCCGTCGGGGCGTGATACCGGCGGCGCGGCCGCCGCCCCGGCGTGGCGAGACCAG |
| H87 | GTTCTTGATACTTCCGTCGCCGCAAACGCCTATTAGAAGACAAGATTGCAGCTGCGAGCCACAGGGTAACAAATTGCTAAACCCTAATATGTTAGCCTGGTGT-CGACAATAGAACGGAATAACCTTGCGGGTGACGGGGCGACGGGGCCGGCGCCGCCGCCGCCGTCGGGGCGTGAT------------------GCCCCGGCGTGGCGAGACCGG |
| H88 | GTCCTTGATACTTCCGTCGCCGCAAACGC------------TAGGTTGCGGCTGCGAGCCATAGGGTAACAAATTGCTAAACCCTAATATGTTAGCCTGGTTT-CGACAATAGAACGGAATAACCTTGCGGGTGACGGGGCGACGGGGCCGGCGCCGCCGCCGCCGTCGGGGCGTGAT------------------GCCCCGGCGCGGTGAGACCGG |
| H89 | GTCCTTGATACTTCCGTCGCCGCAAACGCCTATTAGAAGGCGAGATTGCAGCTGCGAGCCACAGGGTAACAAATTGCTAAACCCTAATATGTTAGCCTGGTGT-CGACAATAGAACGGAATAACCTTGCGGGTGACGGGGCGACGGGGCCGGCGCCGCCGCCGCCGTCGGGGCGTGAT------------------GCCCCGGCGCGGTGAGACCGG |
| H90 | GTCCTTGATAGTTCCGTCGCCGCAAACGCCTATTAGAAGACAAGATTGCGGCTGCGAGCCACAGGGTAACAAATTGCTAAACCCTAATATGTTAGCCTGGTGT-CGACAATAGAACGGAATAACCTTGCGGGTGACGGGGCGACGGGGCCGGCGCCGCCGCCGCCGTCGGGGCGTGAT------------------GCCCCGGCGCGGTGAGACCGG |
| H91 | GTCCTTGATAGTTCCGTCGCCGTAAACGCCTATTAAAAGACGAGGTTGCGACTCCGAGCCATAGGGTAACAAATTGCTAAACCCTAATATGTTAGCATGGTGT-CGACAATAGAACGGAATAACCTTCCGGGTGCCGGGGCGACGGGGCCGGCGCCGCCGCCGCCGTCGGGGCGCGATACCGGCGGCGCGGCCGCCGCCCCGGCGTGGCGAAACCGG |
| H92 | GTTCTTGATACTTCCATCGCCGCAAACGC------------GAGGTTGCGGCTGCGAGCCATAGGGTAACAAATTGCTAAACCCTAATATGTTAGCCTGGTGT-CGACAATAGAACGGAATAACCTTGCGGGTGCCGGGGCGACGGGGCCGGCGCCGCCGCCGCCGTCGGGTCGTGATACCGGCGGCGCGGCCGCCGCCCCGGCGTGGCGAGACCAG |
| H93 | GTTCTTGATACTTCCATCGCCGTAAACGCCTATTAAAAGACGAGGTTGCGACTCCGAGCCATAGGGTAACAAATTGCTAAACCCTAATATGTTAGCCTGGTGT-CGACAATAGAACGGAATAACCTTGCGGGTGCCGGGGCGACGGGGCCGGCGCCGCCGCCGCCGTCGAGTCGTGATACCGGCGGCGCGGCCGCCGCCCCGGCGTGGCGAGACCGG |
| H94 | GTCCTTGATAGTTCCGTCGCCGCAAATGC------------GAAGTTGCAGCTGCGAGCCACAGGGTAACAAATTGCTAAACCCTAATATGTTAGCCTGGTGT-CGACAATAGAACGGAATAACCTTGCCGGTGACGGGGCGACGGGGCCGGCGCCGCCGCCGCCGTCGGGGCGTGAT------------------GCCCCGGCGTGGCGAGACCGG |
| H95 | GTTCTTGATACTTCCATCGCCGTAAACGCCTATTAAAAGACGAGGTTGCGACTCCGAGCCATAGGGTAACAAATTGCTAAACCCTAATATGTTAGCCTGGTGT-CGACAATAGAACGGAATAACCTTCCGGGTGACGGGGCGACGGGGCCGGCGCCGCCGCCGCCGTCGGGTCGTGATACCGGCGGCGCGGCCGCCGCCCCGGCGTGGCGAGACCGG |
| H96 | GTCCTTGATAGTTCCGTCGCCGCAAACGCCTATTAGAAGACAAGATTGCAGCTGCGAGCCACAGGGTAACAAATTGCTAAACCCTAATATGTTAGCCTGGTGT-CGACAATAGAACGGAATAACCTTGCGGGTGCCGGGGCGACGGGGCCGGCGCCGCCGCCGCCGTCGGAGCCTGATACCGGCGGCGCGGCCGCCGCCCCGGCGTGGCGAGACCGG |
| H97 | GTCCTTGATCCTTCCGTCGCCGCAAACACCTATTAAAAGACGAGGTTGTGGCTGCGAGCCATAGGGTAACAAATTGCTAAACCCTAATATGTTAGCCTGGTGT-CGACAATAGAACGGAATAACCTTGCGGGTGCCGGGGCGACGGGGCCGGCGCCGCCGCCGCCGTCGGAGCCTGATACCGGCGGCGCGGCCGCCGCCCCGGCGTGGCGAGACCGG |
| H98 | GTCCTTCATACTTCCGTCGCCGCAAACGTCTATTAAAAGACGAGGTTGTGGCTGCGAGC----------------------C--------------CCGGTGT-CGACAATAGAACGAAATAACCTTGCGGGTGCCGGGGCGACGGGGCCTGCGCCGCCGCCGCCGTCGGGGCGTGATACCGGCGGCGCGGCCGCCGCCCCGGCGTGCCGAGACCGT |
| H99 | GTTCTTGATACTTCCATCGCCGCAAACGCCTATTAAAAGACGAGGTTGCGGCTCCGAGCCATAGGGTAACAAATTGCTAAACCCTAATATGTTAGCATGGTGT-CGACAATAGAACGGAATAACCTGGCGGGTGCCGGGGCGACGGGGCCGGCGCCGCCGCCGCCGTCGGGGCGTGATACCGGCGGCGCGGCCGCCGCCCCGGCGTGGCGAGACCGG |
| H100 | GTCCTTGATACTTCCGTCGCCGCAAACGCCTATTAGAAGACAAGATTGCGGCTGCGAGCCACAGGGTAACAAATTGCTAAACCCTAATATGTTAGCCTGGTGT-CGACAATAGAACGGAATAACCTTGCGGGTGCCGGGGCGACGGGGCCGGCGCCGCCGCCGCCGTCGGGTCGTGAT------------------GCCCCGGCGCGGCGAGACCGG |
| H101 | TTTCTTGATACTTCCATCGCCACAAACGCCTATTAAAAGACGAGGTTGCGGCTGCGAGCCATAGGGTAACAAATTGCTAAACCCTAATATGTTAGCATGGCGT-CGACAATAGAACGGAATAACCTTGCGGGTGCCGGGGCGACGGGGCAGGCGCCGCCGCCGCCGTCGGGGCGTGATACCGGCGGCGCGGCCGCCGCCCCGGCGTGGCGAGGCCGG |
| H102 | TTTCTTGATACTTCCGTCGCCGCAAACG-CTATTAAAAGACGAGATTGCGGCTGCGAGCCACAGGGTAACAAATTGCTAAACCCTAATATGTTAGCCTGGTGT-CGACAATAGAACGGAATAACCTTGCGGGTACCGGGGCGACGGGGCCGGCGCCGCCGCCGCCGTCGGGGCGTGATACCGGCGGCGCGGCCGCCGCCCCGGCGTGGCGAGACCGG |
| H103 | GTTCTTGATACTTCCGTCGCCGCAAACGCCTATTAGAAGACGAGATTGCAGTTGCGAGCCACAGGGTAACAAATTGCTAAACCCTAATATGTTAGCCTGGTGTJCGACAATAGAACGGAATAACCTTGCGGGTGCCGGGGCGACGGGGCCGGCGCCGCCGCCGCCGTCGGGGCGTGAT------------------GCCCCGGCGTGGCGAGACCGG |
| H104 | GTTCTTGATACTTCCATCGCCGTAAACGCCTATTAAAAGACGAGGTTGCGACTCCGAGCCATAGGGTAACAAATTGCTAAACCCTAATATGTTAGCATGGTGT-CGACAATAGAACGGAATAACCTTCCGGGTGCCGGGGCGACGGGGCCGGCGCCGCCGCCGCCGTCGGGTCGTGATACCGGCGGCGCGGCCGCCGCCCCTGCGTGGCGAAACCGG |
| H105 | GTTCTTGATACTTCCATCGCCGTAAACGCCTATTAAAAGACGAGGTTGCGACTCCGAGCCATAGGGTAACAAATTGCTAAACCCTAATATGTTAGCATGGTGT-CGACAATAGAACGGAATAACCTTCCGGGTGCCGGGGCGACGGGGCCGGCGCCGCCGCCGCCGTCGGGTCGTGATACCGGCGGCGCGGCCGCCGCCCCGTCGTGGCGAAACCGG |
|  |  |
| Note: |  |
| J = ACTGCTCT | |
