# Supplementary material for: Sequence polymorphisms in wild, weedy, and cultivated rice suggest seed-shattering locus sh4 played a minor role in Asian rice domestication
Source: Ecol Evol. 2012 Jul 24;2(9):2106–13. doi: 10.1002/ece3.318 (PMC3488663; doi:10.1002/ece3.318)
Supplement: Supplementary file 3 [file ece30002-2106-SD3.doc]

**Appendix 3**. Haplotypes detected in wild, weedy and cultivated rice with their geographic origin and seed shattering phenotype

| Taxon | Haplotype | No. of accession | Countries of origin | Phenotype of seed shattering/persistence |
| --- | --- | --- | --- | --- |
| Wild rice  (166 accessions) | H1 | 26 (15.7%) | Nepal, China, Cambodia, Thailand, India, Laos, Myanmar, Vietnam | Seed shattering |
|  | H2 | 5 (3.0%) | Nepal, Bangladesh, India | Seed shattering |
|  | H3 | 4 (2.4%) | Myanmar, China | Seed shattering |
|  | H4 | 3 (1.8%) | India | Seed shattering |
|  | H5 | 3 (1.8%) | Thailand, China | Seed shattering |
|  | H6 | 3 (1.8%) | Papua New Guinea, Indonesia | Seed shattering |
|  | H7 | 3 (1.8%) | Thailand, Indonesia, Bangladesh | Seed shattering |
|  | H8 | 3 (1.8%) | China, Vietnam, India | Seed shattering |
|  | H9 | 4 (2.4%) | Sri Lanka | Seed shattering |
|  | H10 | 1 (0.6%) | Indonesia | Seed shattering |
|  | H11 | 1 (0.6%) | Nepal | Seed shattering |
|  | H12 | 1 (0.6%) | Sri Lanka | Seed shattering |
|  | H13 | 1 (0.6%) | China | Seed shattering |
|  | H14 | 1 (0.6%) | Indonesia | Seed shattering |
|  | H15 | 1 (0.6%) | Sri Lanka | Seed shattering |
|  | H16 | 1 (0.6%) | India | Seed shattering |
|  | H17 | 1 (0.6%) | India | Seed shattering |
|  | H18 | 2 (1.2%) | India, Nepal | Seed shattering |
|  | H19 | 1 (0.6%) | China | Seed shattering |
|  | H20 | 1 (0.6%) | China | Seed shattering |
|  | H21 | 1 (0.6%) | Thailand | Seed shattering |
|  | H22 | 1(0.6%) | China | Seed shattering |
|  | H23 | 1 (0.6%) | Nepal | Seed shattering |
|  | H24 | 1 (0.6%) | Myanmar | Seed shattering |
|  | H25 | 2 (1.2%) | Sri Lanka, India | Seed shattering |
|  | H26 | 1 (0.6%) | Thailand | Seed shattering |
|  | H27 | 1 (0.6%) | Thailand | Seed shattering |
|  | H28 | 1 (0.6%) | Myanmar | Seed shattering |
|  | H29 | 1 (0.6%) | Myanmar | Seed shattering |
|  | H30 | 1 (0.6%) | Cambodia | Seed shattering |
|  | H31 | 1 (0.6%) | Cambodia | Seed shattering |
|  | H32 | 1 (0.6%) | Indonesia | Seed shattering |
|  | H33 | 1 (0.6%) | China | Seed shattering |
|  | H34 | 2 (1.2%) | China | Seed shattering |
|  | H35 | 1 (0.6%) | China | Seed shattering |
|  | H36 | 1 (0.6%) | China | Seed shattering |
|  | H37 | 2 (1.2%) | China | Seed shattering |
|  | H38 | 1 (0.6%) | Nepal | Seed shattering |
|  | H39 | 1 (0.6%) | Nepal | Seed shattering |
|  | H40 | 1 (0.6%) | India | Seed shattering |
|  | H41 | 1 (0.6%) | Sri Lanka | Seed shattering |
|  | H42 | 1 (0.6%) | Sri Lanka | Seed shattering |
|  | H43 | 1 (0.6%) | Myanmar | Seed shattering |
|  | H44 | 2 (1.2%) | India | Seed shattering |
|  | H45 | 2 (1.2%) | Bangladesh | Seed shattering |
|  | H46 | 2 (1.2%) | India | Seed shattering |
|  | H47 | 2 (1.2%) | Cambodia | Seed shattering |
|  | H48 | 1 (0.6%) | Laos | Seed shattering |
|  | H49 | 1 (0.6%) | Sri Lanka | Seed shattering |
|  | H50 | 1 (0.6%) | Cambodia | Seed shattering |
|  | H51 | 1 (0.6%) | Laos | Seed shattering |
|  | H52 | 1 (0.6%) | Laos | Seed shattering |
|  | H53 | 1 (0.6%) | Cambodia | Seed shattering |
|  | H54 | 1 (0.6%) | Laos | Seed shattering |
|  | H55 | 2 (1.2%) | Nepal | Seed shattering |
|  | H56 | 1 (0.6%) | Nepal | Seed shattering |
|  | H57 | 1 (0.6%) | Sri Lanka | Seed shattering |
|  | H58 | 2 (1.2%) | India | Seed shattering |
|  | H59 | 2 (1.2%) | Laos | Seed shattering |
|  | H60 | 2 (1.2%) | Cambodia | Seed shattering |
|  | H61 | 1 (0.6%) | Myanmar | Seed shattering |
|  | H62 | 1 (0.6%) | Thailand | Seed shattering |
|  | H63 | 1 (0.6%) | Thailand | Seed shattering |
|  | H64 | 1 (0.6%) | Indonesia | Seed shattering |
|  | H65 | 2 (1.2%) | Thailand | Seed shattering |
|  | H66 | 1 (0.6%) | Indonesia | Seed shattering |
|  | H67 | 2 (1.2%) | China | Seed shattering |
|  | H68 | 1 (0.6%) | India | Seed shattering |
|  | H69 | 1 (0.6%) | India | Seed shattering |
|  | H70 | 1 (0.6%) | Thailand | Seed shattering |
|  | H71 | 1 (0.6%) | India | Seed shattering |
|  | H72 | 1 (0.6%) | India | Seed shattering |
|  | H73 | 1 (0.6%) | Indonesia | Seed shattering |
|  | H74 | 1 (0.6%) | Indonesia | Seed shattering |
|  | H75 | 1 (0.6%) | Bangladesh | Seed shattering |
|  | H76 | 2 (1.2%) | Laos, India | Seed shattering |
|  | H77 | 1 (0.6%) | China | Seed shattering |
|  | H78 | 1 (0.6%) | China | Seed shattering |
|  | H79 | 1 (0.6%) | China | Seed shattering |
|  | H80 | 1 (0.6%) | Philippines | Seed shattering |
|  | H81 | 2 (1.2%) | India | Seed shattering |
|  | H82 | 1 (0.6%) | India | Seed shattering |
|  | H83 | 1 (0.6%) | Nepal | Seed shattering |
|  | H84 | 1 (0.6%) | China | Seed shattering |
|  | H85 | 1 (0.6%) | Thailand | Seed shattering |
|  | H86 | 1 (0.6%) | Myanmar | Seed shattering |
|  | H87 | 1 (0.6%) | Thailand | Seed shattering |
|  | H88 | 1 (0.6%) | Laos | Seed shattering |
|  | H89 | 1 (0.6%) | Myanmar | Seed shattering |
|  | H90 | 1 (0.6%) | Vietnam | Seed shattering |
|  | H91 | 1 (0.6%) | Malaysia | Seed shattering |
|  | H92 | 1 (0.6%) | Philippines | Seed shattering |
|  | H93 | 1 (0.6%) | India | Seed shattering |
|  | H94 | 1 (0.6%) | Thailand | Seed shattering |
|  | H95 | 1 (0.6%) | Cambodia | Seed shattering |
|  | H96 | 1 (0.6%) | Vietnam | Seed shattering |
|  | H97 | 1 (0.6%) | China | Seed shattering |
|  | H98 | 1 (0.6%) | Papua New Guinea | Seed shattering |
|  | H99 | 2 (1.2%) | Sri Lanka | Seed shattering |
|  | H100 | 1 (0.6%) | Cambodia, Myanmar | Seed shattering |
|  | H101 | 1 (0.6%) | Vietnam | Seed shattering |
|  | H102 | 1 (0.6%) | China | Seed shattering |
|  | H103 | 2 (1.2%) | India | Seed shattering |
| Weedy rice  (222 accessions) | H1 | 177 (79.7%) | USA, India, Vietnam, Cambodia, Philippines, Nepal, Malaysia, Sri Lanka, Thailand, Laos, Korea, North Korea, Myanmar, Bangladesh, Japan, Bhutan, China | Seed shattering |
|  | H2 | 17 (7.7%) | USA, India, Nepal, Korea, North Korea, Bangladesh, Brazil, China | Seed shattering |
|  | H104 | 24 (10.8%) | Italy, Spain | Seed shattering |
|  | H105 | 4 (1.8%) | USA | Seed shattering |
| Cultivated rice  (192 accessions) | H1 | 160 (83.3%) | Laos, China, Sri Lanka, Cote d'Ivoire, Italy, Iran, India, Bangladesh, Cambodia, Sri Lanka, Thailand, Vietnam, Nepal, USA, Indonesia, Japan, Philippines, Malaysia, Madagascar, Bhutan, Brazil, France, Pakistan, Korea, Nigeria | Seed persistence |
|  | H2 | 32 (16.7%) | Afghanistan, Japan, China, Italy, Nigeria, USA, Australia, Japan, Philippines | Seed persistence |
